# Neural Correlates of Own- and Other-Face Perception in Body Dysmorphic Disorder

**DOI:** 10.3389/fpsyt.2020.00302

**Published:** 2020-04-24

**Authors:** Viktoria Ritter, Jürgen M. Kaufmann, Franziska Krahmer, Holger Wiese, Ulrich Stangier, Stefan R. Schweinberger

**Affiliations:** ^1^ Department of Clinical Psychology and Psychotherapy, Institute of Psychology, Goethe University, Frankfurt, Germany; ^2^ Department of General Psychology and Cognitive Neuroscience, Institute of Psychology, Friedrich Schiller University, Jena, Germany; ^3^ Department of Psychology, Durham University, Durham, United Kingdom

**Keywords:** body dysmorphic disorder, own-face perception, face inversion effect, event-related potentials, electroencephalography

## Abstract

**Background:**

Body dysmorphic disorder (BDD) is characterized by an excessive preoccupation with one or more perceived flaws in one’s own appearance. Previous studies provided evidence for deficits in configural and holistic processing in BDD. Preliminary evidence suggests abnormalities at an early stage of visual processing. The present study is the first examining early neurocognitive perception of the own face in BDD by using electroencephalography (EEG). We investigated the face inversion effect, in which inverted (upside-down) faces are disproportionately poorly processed compared to upright faces. This effect reflects a disruption of configural and holistic processing, and in consequence a preponderance of featural face processing.

**Methods:**

We recorded face-sensitive event-related potentials (ERPs) in 16 BDD patients and 16 healthy controls, all unmedicated. Participants viewed upright and inverted (upside-down) images of their own face and an unfamiliar other face, each in two facial emotional expressions (neutral vs. smiling). We calculated the early ERP components P100, N170, P200, N250, and the late positive component (LPC), and compared amplitudes among both groups.

**Results:**

In the early P100, no face inversion effects were found in both groups. In the N170, both groups exhibited the common face inversion effects, with significantly larger N170 amplitudes for inverted than upright faces. In the P200, both groups exhibited larger inversion effects to other (relative to own) faces, with larger P200 amplitudes for other upright than inverted faces. In the N250, no significant group differences were found in face processing. In the LPC, both groups exhibited larger inversion effects to other (relative to own) faces, with larger LPC amplitudes for other inverted than upright faces. These overall patterns appeared to be comparable for both groups. Smaller inversion effects to own (relative to other) faces were observed in none of these components in BDD, relative to controls.

**Conclusions:**

The findings suggest no evidence for abnormalities at all levels of early face processing in our observed sample of BDD patients. Further research should investigate the neural substrates underlying BDD symptomatology.

## Introduction

A large body of research supports that faces are processed holistically in healthy humans (1, 2). Research indicates that the perception of a specific part is not independent of other parts, and that faces are processed as a gestalt whole (3, 4). Clinical researchers showed heightened interest in abnormalities in holistic face processing in psychiatric disorders. Body dysmorphic disorder (BDD) is characterized by an excessive preoccupation with perceived defect(s) or flaws in one’s own physical appearance which are not observable or appear slight to others (5). This preoccupation often pertains to the own face in particular. Individuals with BDD focus on details in the appearance of their skin, hair, or other facial parts (6). They believe that these features are disfigured or ugly and often have delusional beliefs (7). Some individuals experience high levels of suffering and distress. BDD is often accompanied by high psychiatric comorbidity (8), suicidality (9), low quality of life, and can result in severe psychosocial impairments (10).

Neuropsychological and cognitive-behavioral models emphasize the potential role of a detailed perception in the maintenance of BDD [e.g., (11–13)]. Behavioral studies with different experimental paradigms using own-face (14), own- and other-face (15, 16), and other-face (17) stimuli provided evidence for a high aestethic sensitivity, enhanced discrimination abilities [but see (18, 19)], and a selective attention toward perceived flaws in BDD [e.g., (20, 21)]. Neuroimaging studies indicate a detailed processing of faces and an aberrant processing of configural and holistic information in BDD (22, 23). However, it remains unclear whether the abnormal brain activation patterns are primarily the result of an aberrant early visual cortex activity or of a modulation by prefrontal and/or limbic systems (24). In an electroencephalography (EEG) study, evidence was found for significantly smaller N170 amplitudes in BDD, compared to healthy controls, for normal images, regardless of stimulus type (faces or houses) (24). There was also a trend for longer N170 latencies, regardless of spatial frequency or stimulus type. These findings may suggest perceptual abnormalities at an early stage of visual processing in BDD. A later study assessing EEG and fMRI (25) found that compared to healthy controls, individuals with BDD and anorexia nervosa demonstrated similar hypoactivity in early secondary visual processing regions including lateral occipital cortex (linked to the N170 component), occipital pole and precuneus for low spatial frequency (i.e., low-detail) faces, indicating abnormal spatiotemporal activation of configural and holistic information in BDD.

A paradigm providing insight into configural and holistic face processing is the face inversion effect (26). Previous research suggests that upright faces are processed with the use of configural information by forming a holistic face representation, whereas inverted faces tend to be processed by using featural information in face-sensitive brain regions (27, 28). In healthy humans, inverting a face disrupts configural and holistic face processing, and has no (or less) influence on the processing of featural information (29). Four behavioral studies investigated the face inversion effect in BDD, yielding inconsistent results. Whereas Feusner et al. (30) and Mundy and Sadusky (31) found smaller inversion effects, and Jeffries et al. (32) a superior recognition of inverted famous faces in BDD, relative to controls, all suggesting a greater detailed processing, no differences were found by Monzani et al. (33). However, different experimental conditions (e.g., presentation time), variation in stimuli (e.g., familiarity), sample characteristics (e.g., different degrees of BDD severity) or insufficient statistical power might explain the inconsistent findings.

Building on previous studies and using an established paradigm (34), we neurophysiologically determined whether or not any potential face inversion effects are specific to own faces in BDD. Smaller face inversion effects to own faces would be in line with the interpretation that holistic processing of own faces is specifically compromised in BDD. We assessed the following early ERP components [cf. (35)]: (1) The occipito-temporal P100 is related to the processing of low-level physical characteristics (36), occurs about 80–120 ms poststimulus (37), and was assessed to investigate processes preceding face detection and structural encoding. (2) The occipito-temporal N170 is sensitive to face inversion, with larger amplitudes and delayed latencies for inverted than upright faces [e.g., (38, 39)], and occurs about 150–180 ms poststimulus. Larger N170 amplitudes for inverted faces reflect a disruption of configural processing and a preponderance of featural processing. The N170 is modulated by “self-information,” and more negative for own relative to other faces [e.g., (40, 41)]. (3) The occipito-temporal P200 occurs about 200–250 ms poststimulus, has been related to the perceived typicality of a face [e.g., (40, 42, 43)], and is larger for less distinctive (i.e., more typical) faces. (4) The occipito-temporal N250 component is responsive to face familiarity [e.g., (44, 45)], occurs about 260–400 ms poststimulus. (5) The centro-parietal late positive component (LPC) occurs about 400–600 ms poststimulus, and has been related to the activation and recognition of emotional content of stimuli [e.g., (46)].

In line with previous research in BDD (24, 25), on the P100, we hypothesized smaller P100 amplitudes in BDD, compared to healthy controls, which would reflect abnormal early configural processing. On the N170 component, we predicted smaller N170 inversion effects to own (relative to other) faces in BDD, compared to healthy controls, with smaller amplitudes for own inverted than upright faces, as we hypothesized predominantly featural processing of own faces not only in inverted but already in upright faces in BDD. On the P200 component, we expected smaller P200 inversion effects to own (relative to other) faces in BDD, compared to healthy controls, with smaller P200 amplitudes for own inverted than upright faces, which would reflect predominantly featural processing and a lower perceived typicality of the own face (i.e., the own face is perceived as less typical and more distinctive) in BDD. In the N250 component, we expected smaller N250 inversion effects to own (relative to other) faces in BDD, compared to controls, and larger N250 amplitudes for own (relative to other) faces in BDD, which would reflect a higher familiarity with the own face that may result from a permanent preoccupation with perceived defects in the own face. In the LPC component, we expected smaller LPC inversion effects to own (relative to other) faces in BDD, compared to controls, and larger LPC amplitudes for own (relative to other) faces, which would reflect a less efficient emotional recognition of own faces in BDD. Dependent variables were the amplitudes on the P100, N170, P200, N250, and the LPC components.

## Materials and Methods

### Participants

The EEG study was part of a research program in which we also investigated adaptive face coding mechanisms in BDD (results are reported elsewhere). Participants were recruited between 2012 and 2015 *via* an outpatient unit at Goethe University Frankfurt, Germany, and *via* flyers that we posted in coffee shops, at university, or libraries, and sent to psychotherapists, plastic surgeons, and dermatologists for distribution to their patients. All participants were age- and gender-matched. BDD patients had a primary diagnosis of BDD confirmed by a licensed clinical psychologist (VR) administering the German version of the Structured Clinical Interview for DSM-IV-TR [SCID; (47, 48)], the German version of the clinician-administered BDD Diagnostic Module [BDDDM; (49, 50)], and the German version of the clinician-administered BDD Modification of the Yale-Brown Obsessive-Compulsive Scale [BDD-YBOCS; (49, 51)]. Given that the BDD-YBOCS was developed for clinical samples of BDD patients (52), the measure was administered to BDD participants only. The following self-report measures were applied: the Body Dysmorphic Symptoms Inventory [FKS; Fragebogen Körperdysmorpher Symptome; (53)], the German version of the Beck Depression Inventory-II [BDI-II; (54, 55)], and the German version of the Brief Symptom Inventory [BSI; (56, 57)].

### BDD Inclusion/Exclusion Criteria

The study protocol was approved by the ethics committee of the Medical Faculty of the Goethe University Frankfurt (Ref. No. 39/11) and conducted in accordance with the declaration of Helsinki. Written informed consent was obtained from all participants. Individuals who met diagnostic criteria for BDD as determined by the BDDDM, and who scored higher or equal to 20 on the BDD-YBOCS (52) were eligible. In order to comprise a clinically representative sample, all concurrent Axis I disorders less severe than BDD were permitted except those listed among the following exclusion criteria: current or past obsessive-compulsive and related disorders, a history of psychotic or bipolar disorders, suicidality, concurrent psychotherapeutic, and psychopharmacological treatment.

### HC Inclusion/Exclusion Criteria

Healthy controls showed no current or past Axis-I psychiatric history, as determined by the SCID. All participants (BDD and HC) had normal or corrected to normal vision and all but one was right-handed as determined by the Edinburgh Inventory (58).

### Stimuli and Apparatus

Stimuli consisted of 10 different images of the own face and 10 different images of an unfamiliar gender- and age-matched face, which were prepared under standardized conditions using a digital camera. The 10 images showed each face in five different orientations: frontal view, 22.5° left or right side and 45° left or right side; this was done to increase variability of stimuli and to discourage a processing strategy that focusses on individual images, rather than faces. Each orientation was shown with either neutral or smiling expressions. The facial emotional expression “smiling” was chosen to increase the number of stimuli, and to take into account previous research which found biases in processing of facial emotional expressions in BDD [e.g., (32, 59)]. Where necessary, stimuli were transformed to approximately equal luminance and contrast, and any information from the neck downwards, such as clothing, and accessories were removed. Raw pictures were adjusted to 170 × 260 pixels.

**Figure 1:**
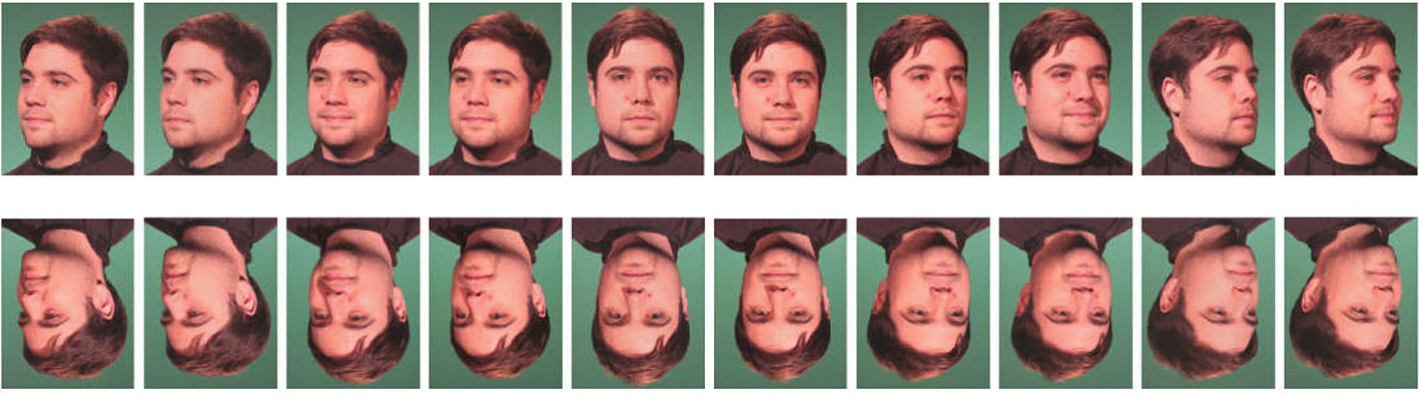
Stimulus examples of one male identity. The images show the 20 different faces (frontal view, 22.5° left and right side, 45° left and right side each with neutral and smiling expression, upright and inverted position).

Overall, 20 images were used for experimental trials, each in upright or inverted position, resulting in 40 different stimuli for experimental trials. In addition, ten images of another gender-matched unfamiliar face (each in the five different orientations, neutral or smiling expression, upright, or inverted) were used as stimuli for 20 practice trials. Stimulus examples are given in **Figure 1**.

All stimuli were presented on a dark gray screen in the center of a 19” Samsung SyncMaster 795DF CFT-monitor. The presentation software was Eprime™ (Version 2). Stimuli were presented at a viewing distance of 90 cm, which was kept constant by using a chin rest (visual angle 5.4° × 6.9°).

### Experimental Procedure

At the beginning of the experiment, participants received written instructions on the screen. They were instructed to decide whether the presented face is either in a veridical (upright) or in an inverted position, by pressing marked keys (“f” and “j”) on a standard keyboard (German layout) using the index fingers of both hands. Note that our rationale for the present task was that we wished ensure that participants had attentively processed the stimuli, while at the same time the task itself should *not* direct participants´ attention to the identity of the face (own or other). Accordingly, we reasoned that any differences in neural processing of own and other face reflected spontaneous processing differences, with no interference from the specific task demands.

**Figure 2:**
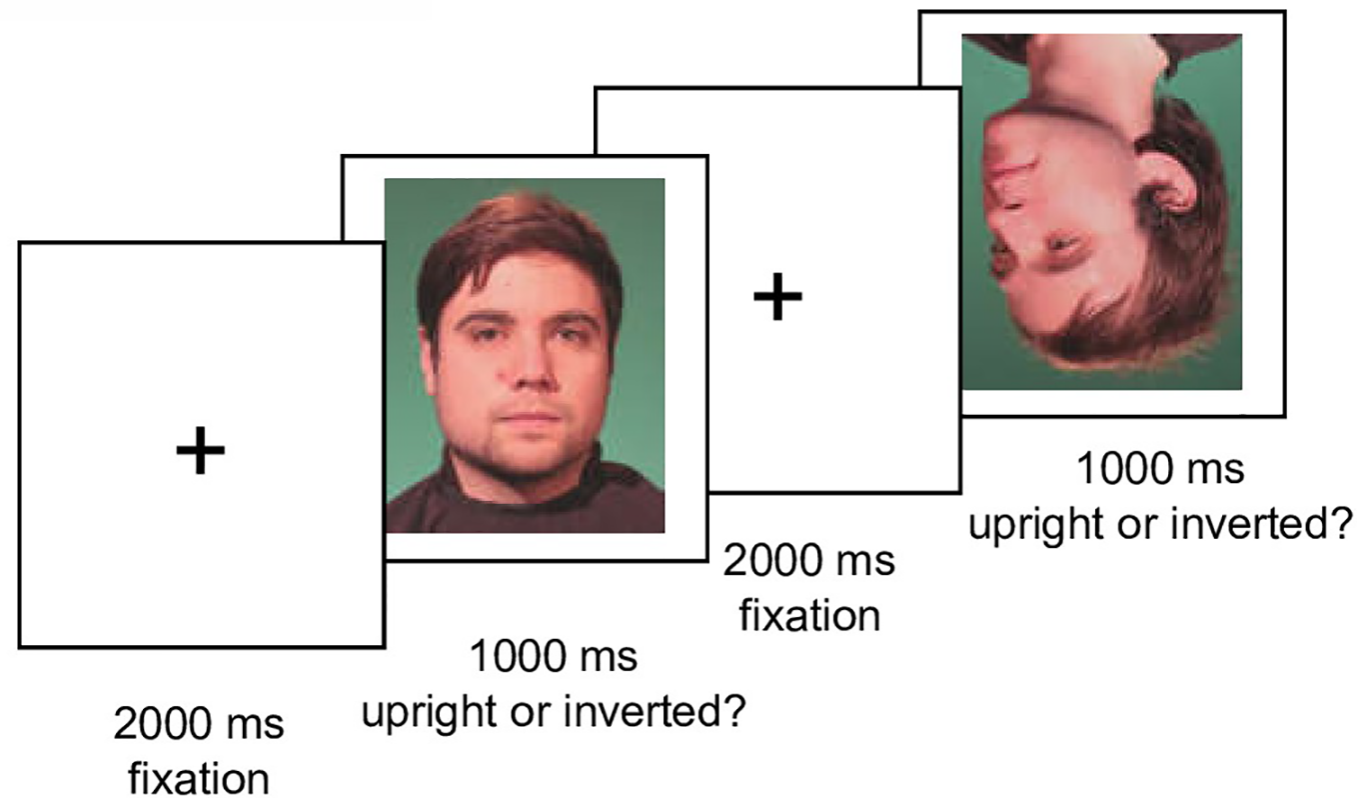
Schematic trial sequence of the experiment. Illustration of two trials with an upright and an inverted male face in the test phase.

The experiment consisted of a practice phase (20 trials) and an immediately following test phase. The test phase consisted of 10 blocks (400 trials), each block containing stimuli of each condition (five different face orientations, upright vs. inverted position, own vs. other face, neutral vs. smiling expression). Within blocks, stimuli were presented in randomized order. Between blocks, participants were allowed a self-paced break. Each trial started with a fixation cross (2,000 ms), followed by a face (1,000 ms) and another fixation cross (2,000 ms) [in accordance with (34)]. A schematic sequence of the experiment is given in **Figure 2**.

### EEG Acquisition

EEG data were recorded in an electrically and acoustically shielded room with sintered Ag/AgCl electrodes mounted in an electrode cap (EasyCap™, Herrsching-Breitbrunn, Germany) using a BioSemi Active II System (BioSemi, Amsterdam, Netherlands). Thirty-two electrodes were arranged according to an extended 10/20 system at the scalp positions Fz, Cz, Pz, Iz, FP1, FP2, F3, F4, C3, C4, P3, P4, O1, O2, F7, F8, T7, T8, P7, P8, F9, F10, FT9, FT10, TP9, TP10, P9, P10, PO9, PO10, I1, and I2. The horizontal electrooculogram (EOG) was recorded from F9’ and F10’ at the outer canthi of both eyes. The vertical EOG was monitored bipolarly from electrodes above and below the right eye. Signals were recorded with DC (120 Hz, −6 dB attenuation, 12 dB/octave), and sampled at 512 Hz.

Offline, ocular artifacts were automatically corrected using BESA™ 5.1 (60). Epochs were generated, lasting 1,200 ms, including a 200-ms prestimulus baseline interval. In the test phase, we analyzed only trials with correct responses. Trials contaminated by nonocular artifacts were rejected from further analysis using the BESA™ artifact rejection tool (amplitude threshold 100 µV, gradient criterion 50 µV). The EEG was low-pass filtered at 40 Hz, and recalculated to average reference, excluding vertical and horizontal EOG channels. Trials were averaged separately for each channel and experimental condition (own and other, upright and inverted, neutral and smiling faces). The average numbers of correct and artifact-free trials over all conditions in the test phase were 44.4 and 43.8 for BDD patients and healthy controls, respectively.

ERPs were quantified using mean amplitudes for the occipito-temporal P100 (80–120 ms), N170 (120–164 ms), P200 (195–225ms) and N250 (250–340ms) components, as well as for a centro-parietal LPC (400–600 ms), all relative to the 200-ms prestimulus baseline. Time intervals were selected based on previous research and visual inspection of the grand mean ERPs. Effects were quantified at selected electrodes of interest, based on maxima of a particular component in grand mean ERPs and previous research [e.g., (34, 61, 62)]: P100 (O1/O2), N170 (P7/P8, P9/P10, PO9/PO10), P200 (O1/O2, P9/P10, PO9/PO10), N250 (P7/P8, P9/P10, PO9/PO10), and LPC (C3/C4, P3/P4, Cz, and Pz).

### Power Analysis

A priori, a power analysis was computed using G-Power 3.1.9.2 (63), with repeated measures ANOVA (within-between design), a moderate effect of f= 0.25 between BDD patients and controls, a power of 0.80 and a correlation among the repeatedly measured dimensions of r = 0.5 (64), resulting in a total sample size of 24. Hence, including a supposed dropout rate of 25% in BDD studies [e.g., (65)], at least a total of 32 participants had to be recruited for this study.

### Statistical Analysis

For behavioral analyses, response times (RTs) were analyzed for correct responses only. Error of omissions (no key press) and responses < 200 ms were excluded from statistical analyses. Response accuracy (% correct) and mean correct RTs were computed for each condition and both groups. Analyses of variance (ANOVAs) were performed, with epsilon corrections for heterogeneity of covariances (66) where appropriate. For behavioral analyses, we performed ANOVAs with repeated measurements on face type (own vs. others), orientation (upright vs. inverted) as within-subject factors, and group (BDD vs. HC) as between-subjects factor. For ERP analyses, we performed ANOVAs with repeated measurements on face type (own vs. others), orientation (upright vs. inverted), expression (neutral vs. smiling), hemisphere (left vs. right; not for LPC), site (electrodes, depending on ERP component) as within-subject factors, and group (BDD vs. HC) as between-subjects factor. To further investigate significant group effects or interactions involving group, we subsequently performed separate ANOVAs for both groups. We included the factor expression because recent research provided evidence for biases in emotional face recognition in BDD [e.g., (32, 59)].

In addition, we investigated in ***post hoc*** Bayesian analysis for both behavioral and ERP data, to test evidence for null results. To estimate support for null hypothesis, Bayes factors for ANOVA designs were computed (67) using the statistical software JASP [JASP, 2019; (68, 69)]. For the interpretation of the Bayesian factors, we followed the cutoff values of 0.3 and 3 proposed by Jeffreys, with values between 0.3 and 3 indicating there is no sufficient empirical evidence for the absence of the observed effects (70). Bayesian factors > 3.0 indicate that the confirmation of null hypothesis has a probability of more than three times higher than the alternative hypothesis.

## Results

**Table 1** summarizes demographic and psychometric data. All participants were unmedicated. BDD and healthy controls did not differ in mean age or educational level. BDD participants were preoccupied with at least one perceived facial defect. Seven patients were additionally preoccupied with perceived bodily defects.

**Table 1:** Sample characteristics by group for demographic, body dysmorphic disorder (BDD)–related and symptom measures.

	Body Dysmorphic Disorder (BDD)	Healthy Controls (HC)	Stats (*t*)	*p*-value
N	16	16		
Gender (F/M)	11/5	11/5		
Age, mean (SD)	26.19 (8.06)	26.13 (8.23)	0.02	0.983
Age range (min, max)	(20, 54)	(20, 55)	*`*	
Highest School Diploma Completed	9/16	9/16		
Age of onset BDD	16.31 (10.64)			
Comorbidities (n)	Major Depressive Disorder (4) Dysthymia (1) Social Anxiety Disorder (3) Panic Disorder with Agoraphobia (1) Loss of Sexual Desire (1)			
BDD-YBOCS	25.06 (2.98)			
FKS	31.88 (7.72)	7.75 (4.04)	11.08	0.000
BDI-II	17.38 (11.49)	5.56 (4.29)	3.85	0.001
BSI (GSI)	66.19 (10.74)	44.44 (8.25)	6.43	0.000

Means and standard deviations in parentheses.

BDD-YBOCS, Yale-Brown Obsessive-Compulsive Scale Modified for BDD; FKS, Body Dysmorphic Symptoms Inventory; BDI-II, Beck Depression Inventory-II; BSI, Brief Symptom Inventory; GSI, Global Severity Index.

### Behavioral Data

#### Reaction Times

**Figure 3:**
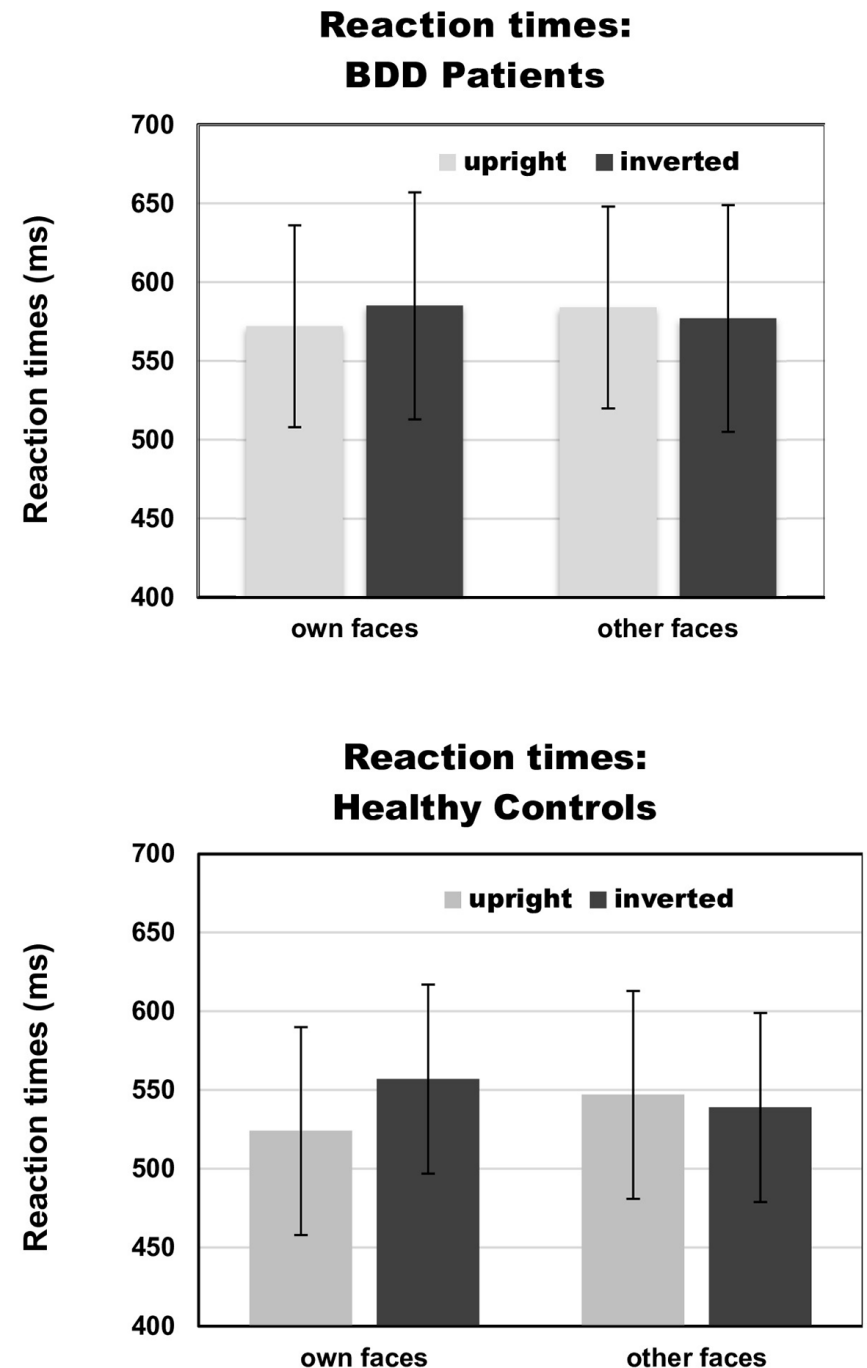
Reaction times on face type (own vs. other) and orientation (upright vs. inverted) by group [body dysmorphic disorder (BDD) patients vs. healthy controls]. Error bars indicate standard deviations.

There was a significant interaction between face type and orientation, *F*(1,30) = 7.65, *p* =.010, *η_p_^2^* =.31, but no significant group effect, *F*(1,30) = 2.88, *p* =.10, *η_p_^2^* =.03, and no significant group interactions that included either the factors face type or orientation, *F*s (1,30) < 1. To follow up on the significant interaction of face type and orientation, we performed pairwise comparisons among face type and orientation. Overall, there were significantly longer reaction times for own inverted (mean reaction time = 571 ms, SD = 67 ms) than own upright faces [mean reaction time = 548 ms, SD = 69 ms, *t*(31) = 3.13, *p* =.004]. Reaction times for other inverted (mean reaction time = 558 ms, SD = 68 ms) and other upright faces [mean reaction time = 566 ms, SD = 75 ms) did not significantly differ, *t*(31) = 0.88, *p* =.386]. See **Table 2** and **Figure 3** for reaction times on face type and orientation by group.

**Table 2:** Accuracies in % for correct responses, reaction times in ms, and between-group multiple comparisons per condition in the test phase for face type, and orientation by group.

	Body Dysmorphic Disorder (BDD)		Healthy controls (HC)		Stats *t*	*p*-value	*t*	*p*-value
	RT	Acc	RT	Acc	RT		Acc	
Own Upright Faces	572 (64)	92.4 (5.1)	524 (66)	95.1 (4.6)	-2.06	0.048	0.88	0.386
Own Inverted Faces	586 (72)	92.5 (6.9)	557 (60)	91.1 (0.1)	-1.22	0.232	-0.15	0.882
Other Upright Faces	584 (74)	91.3 (7.9)	547 (74)	91.0 (8.5)	-1.41	0.169	-0.56	0.581
Other Inverted Faces	577 (72)	93.6 (4.9)	539 (61)	95.5 (4.4)	-1.64	0.111	0.59	0.559

Standard deviations (SD) are given in parentheses.

Acc, accuracy; RT, reaction time.

For the interaction between face type, orientation and group, the Bayesian analysis revealed a BFexcl= 1.925, indicating that there is no sufficient evidence for the absence of this effect. Statistics of Bayesian analysis for main and interaction effects can be found in **Table S1** (**Supplementary Materials**).

#### Accuracy

Overall, response accuracy was generally close to ceiling (BDD: *M* = 0.92, *SD* = 0.05; HC: *M* = 0.93, *SD* = 0.04). There was a significant interaction between face type and orientation, *F*(1,30) = 9.76, *p* =.004, *η_p_^2^* =.38, but no significant group effect, *F*(1,30) = 0.01, *p* =.913, *η_p_^2^* = 0, and no significant interactions including the factor group, *F*s (1,30) < 1.8. To follow up on the significant interaction of face type and orientation, we performed pairwise comparisons among face type and orientation. Overall, response accuracies were higher for own upright (M = 0.94, SD = 0.05) than own inverted faces [M = 0.91, SD = 0.08, *t*(31) = 2.16, *p* =.039], and for other inverted (M = 0.94, SD = 0.06) than other upright faces [M = 0.91, SD = 0.10, *t*(31) = 2.63, *p* =.013]. See **Table 2** for summary of accuracies by group.

For the interaction between face type, orientation and group, the Bayesian analysis revealed a BF_excl_= 1.013, indicating that there is no sufficient evidence for the absence of this effect. Statistics of Bayesian analysis for main and interaction effects can be found in **Table S2** (**Supplementary Materials**).

### ERPs

#### P100 Amplitude (80–120 ms)

We found no significant group effect, *F*(1,30) = 1.68, *p* =.205, *η_p_^2^* =.05, and no significant main effects of face type and orientation, *F*s (1,30) < 0.76. There were no significant group interactions including the factors face type and orientation, *F*s (1,30) < 1.84. Statistics for main and interaction effects can be found in **Table S3** (**Supplementary Materials**).

For the interaction between face type, orientation and group, the Bayesian analysis revealed a BFexcl= 5.260, indicating that there is 5.3 times higher probability for support of the null effect than alternative hypothesis. Statistics of Bayesian analysis for group interaction effects can be found in **Table S4** (**Supplementary Materials**).

#### N170 Amplitude (120–164 ms)

We found no significant group effect, *F*(1,30) = 0.14, *p* =.71, *η_p_^2^* =.01. There was a significant main effect of face type, *F*(1,30) = 4.25, *p* =.048, *η_p_^2^* =.12, and orientation, *F*(1,30) = 6.55, *p* =.016, *η_p_^2^* =.18, but no significant interaction between face type and orientation, *F*(1,30) = 1.46, *p* =.237, *η_p_^2^* =.05. Analyses revealed no significant group interactions including either the factors face type and orientation, *F*s (1,30) < 2.01. There was a trend for an interaction between site, face type, orientation, and group, *F*(2,60) = 3.31, *p* =.058, *η_p_^2^*=.10. Statistics for all main and interaction effects can be found in **Table S5** (**Supplementary Materials**).

**Figure 4:**
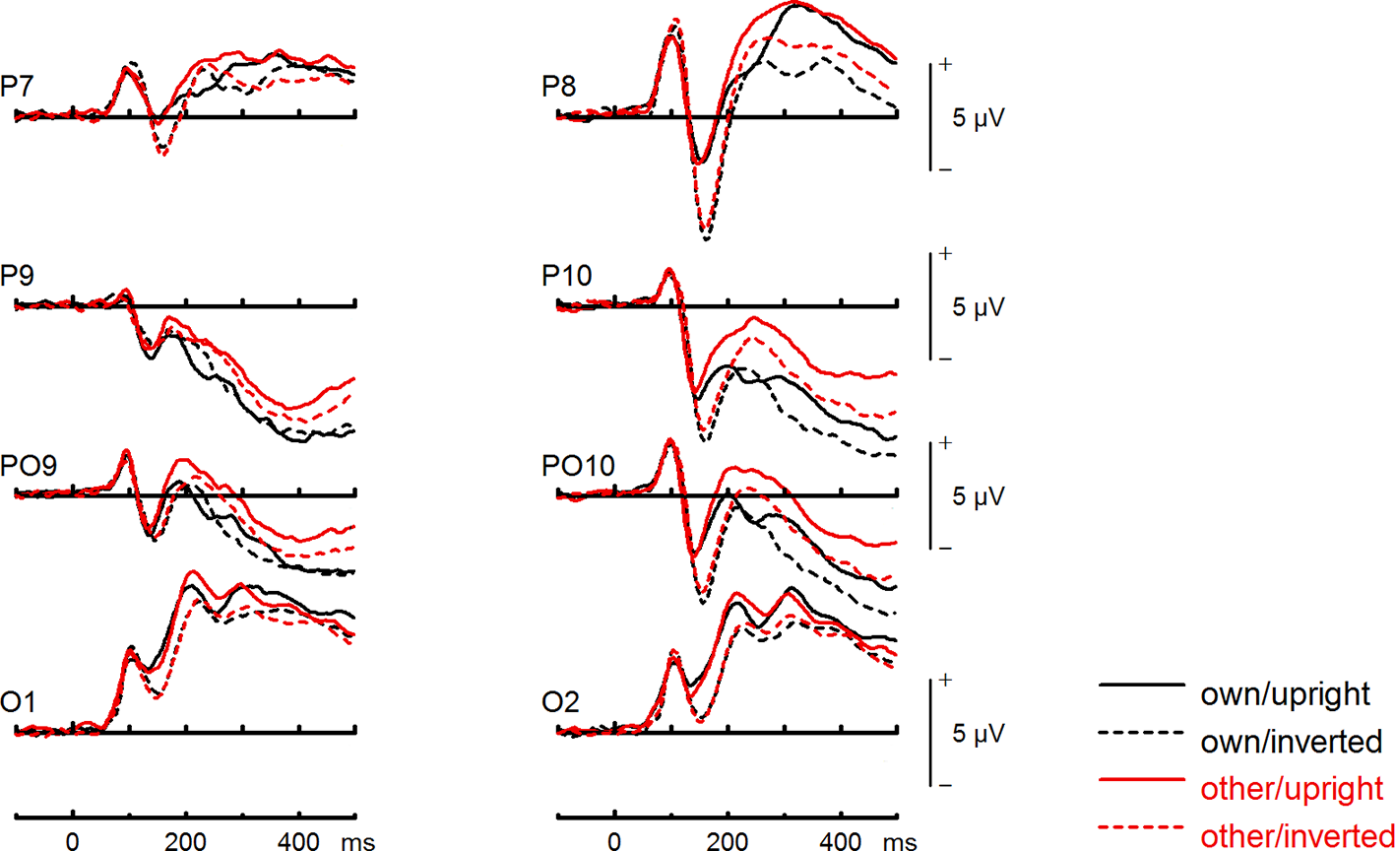
Event-related potentials (ERPs) from the test phase for the interaction face type (own vs. other) by orientation (upright vs. inverted) in body dysmorphic disorder (BDD) patients. At occipito-temporal electrodes (P7/P8, P9/P10, PO9/PO10, O1/O2) within time intervals for P100, N170, P200, and N250.

**Figure 5:**
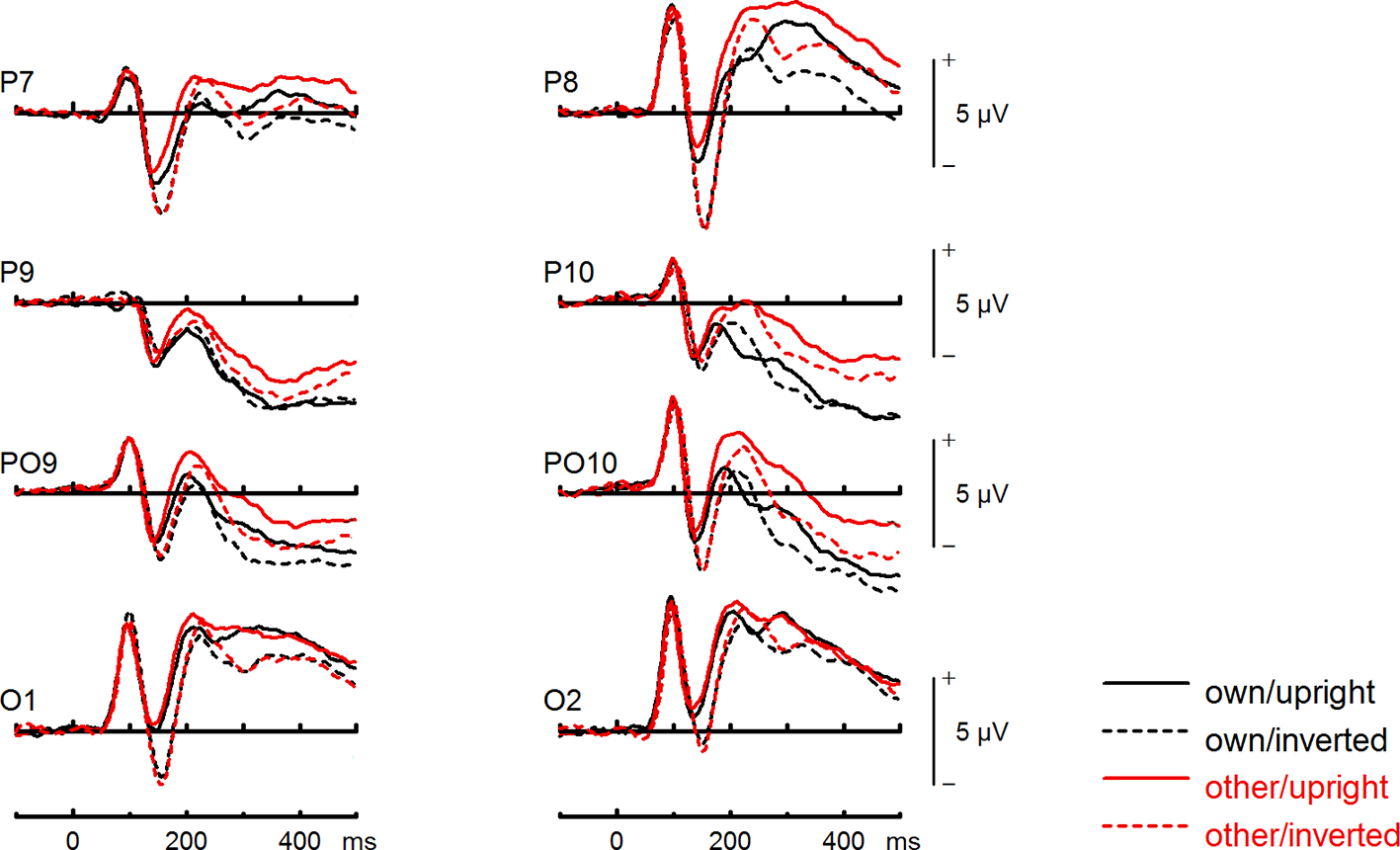
Event-related potentials (ERPs) from the test phase for the interaction face type (own vs. other) by orientation (upright vs. inverted) in healthy controls. At occipito-temporal electrodes (P7/P8, P9/P10, PO9/PO10, O1/O2) within time intervals for P100, N170, P200, and N250.

**Figure 6:**
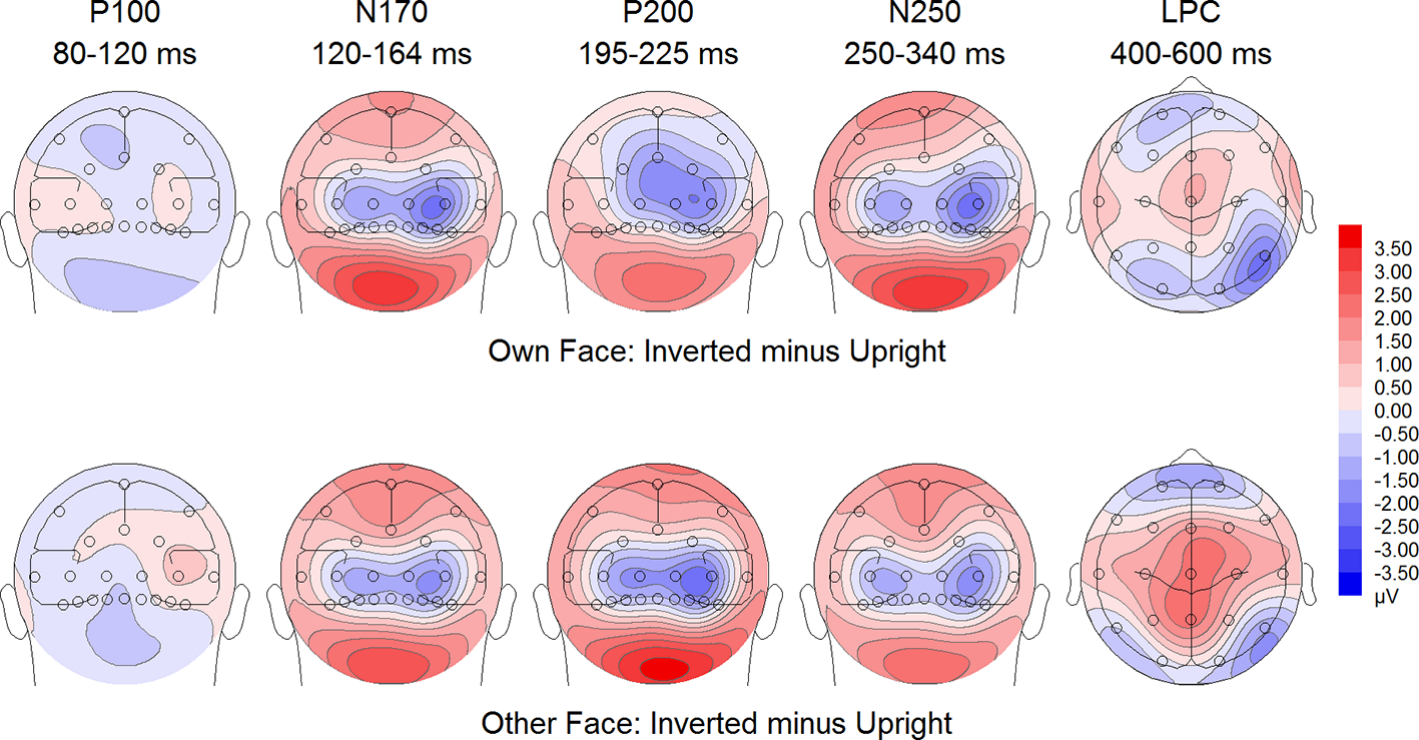
Difference maps from the test phase for the interaction face type (own vs. other) by orientation (upright vs. inverted) in body dysmorphic disorder (BDD) patients. At occipito-temporal electrodes (P7/P8, P9/P10, PO9/PO10, O1/O2) within time intervals for P100, N170, P200, N250 and LPC.

**Figure 7:**
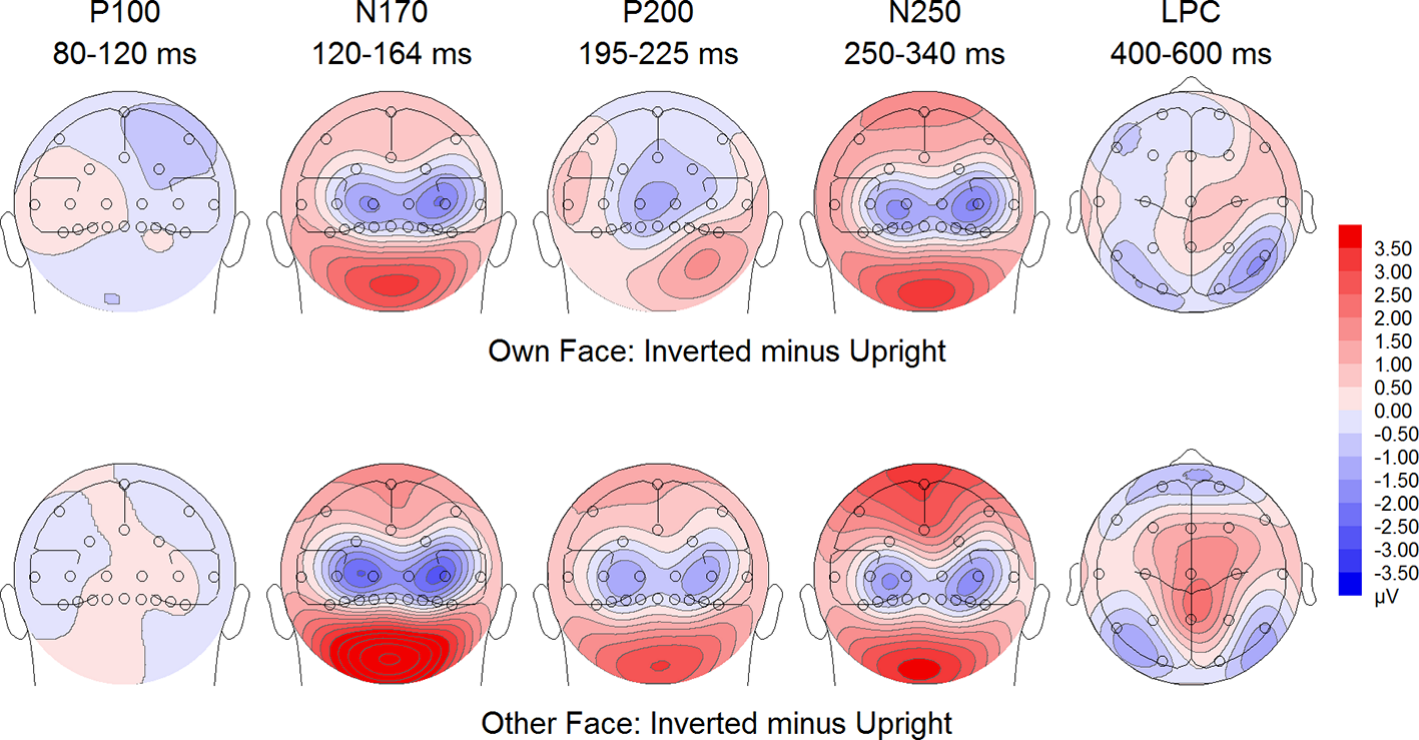
Difference maps from the test phase for the interaction face type (own vs. other) by orientation (upright vs. inverted) in healthy controls. At occipito-temporal electrodes (P7/P8, P9/P10, PO9/PO10, O1/O2) within time intervals for P100, N170, P200, N250 and LPC.

To further investigate the interaction trend, we performed separate ANOVAs for both groups. In BDD, a significant main effect of orientation, *F*(1,15) = 5.92, *p* =.028, *η_p_^2^* =.28, with larger N170 amplitudes for inverted faces and smaller N170 amplitudes for upright faces. A significant main effect of site, *F*(2,30) = 16.85, *p* < .001, *η_p_^2^ =.*53, was further qualified by an interaction with orientation, *F*(2,30) = 3.35, *p* =.049, *η_p_^2^ =.*18, and reflected larger N170 amplitudes for inverted faces at P8, P10 and PO10 electrodes (see **Figure 4**). There was no significant main effect of face type, *F*(1,15) = 0.58, *p* =.457, *η_p_^2^* =.04, and no significant interaction between face type and orientation, *F*(1,15) = 0.02, *p* =.886, *η_p_^2^* =.00 (see **Figure 6**). In HC, a significant main effect of face type, *F*(1,15) = 8.54, *p* =.010, *η_p_^2^* =.36, indicating larger N170 amplitudes for other (relative to own) faces. There was no significant main effect of orientation, *F*(1,15) = 1.97, *p* =.180, *η_p_^2^* =.12, but a significant interaction between site and orientation, *F*(1,15) = 7.18, *p* =.010, *η_p_^2^* =.32, indicating larger N170 amplitudes for inverted faces and smaller N170 amplitudes for upright faces at P8 and PO10 electrodes (see **Figure 5**). Analyses revealed no significant interaction between face type and orientation, *F*(1,15) = 3.65, *p* =.076, *η_p_^2^* =.20 (see **Figure 7**).

For the interaction between face type, orientation and group, the Bayesian analysis revealed a BFexcl= 5.282, indicating that there is 5.3 times higher probability for support of the null effect than alternative hypothesis. Statistics of Bayesian analysis for group interaction effects can be found in **Table S6** (**Supplementary Materials**).

#### P200 Amplitude (195–225 ms)

We found no significant group effect, *F*(1,30) = 0.37, *p* =.55, *η_p_^2^* =.01. There was a significant main effect of face type, *F*(1,30) = 46.09, *p* < .001, *η_p_^2^* =.61, and orientation, *F*(1,30) = 19.38, *p* < .001, *η_p_^2^* =.39. Analyses revealed a significant interaction between face type and orientation, *F*(1,30) = 27.24, *p* < .001, *η_p_^2^* =.48. There were no significant group interactions including the factors face type, and orientation, *F*s(1,30) < 1.99, but a significant interaction between site, face type, orientation, expression, and group, *F*(2,60) = 4.66, *p* =.021, *η_p_^2^* =.13. Statistics for all main and interaction effects can be found in **Table S7** (**Supplementary Materials**).

To further investigate the significant interaction effect, we performed separate ANOVAs for both groups. In BDD, significant main effects of face type, *F*(1,15) = 35.41, *p* = 0, *η_p_^2^* =.70, orientation, *F*(1,15) = 13.12, *p* =.003, *η_p_^2^* =.47. The significant interaction between face type and orientation, *F?A3B2 show $132#?>*(1,15) = 17.61, *p* =.001, *η_p_^2^* =.54, indicated larger P200 inversion effects to other (relative to own) faces, with larger P200 amplitudes for other upright than other inverted faces (see **Figure 6**). The significant interaction between site and face type, *F*(2,30) = 4.35, *p* =.047, *η_p_^2^* =.23, indicated larger P200 amplitudes for other (relative to own) faces at P10 and PO10 electrodes (see **Figure 4**). In HC, significant main effects of face type, *F*(1,15) = 13.77, *p* =.002, *η_p_^2^* =.48, and orientation, *F*(1,15) = 7.26, *p* =.017, *η_p_^2^* =.33. Analyses revealed a significant interaction between face type and orientation, *F*(1,15) = 10.07, *p* =.006, *η_p_^2^* =.40, again indicating larger P200 inversion effects to other (relative to own) faces, with larger P200 amplitudes for other upright than other inverted faces (see **Figure 7** *).* There were significant interactions between site and face type, *F*(2,30) = 3.97, *p* =.044, *η_p_^2^* =.21, as well as site and orientation, *F*(2,30) = 17.50, *p* < .001, *η_p_^2^* =.54, with larger (i.e., more positive) P200 amplitudes for other upright than other inverted faces at O1 and O2 electrodes (**Figure 5**).

For the interaction between face type, orientation and group, the Bayesian analysis revealed a BFexcl= 4.484, indicating that there is 4.5 times higher probability for support of the null effect than the alternative hypothesis. Statistics of Bayesian analysis for group interaction effects can be found in **Table S8** (**Supplementary Materials**).

#### N250 Amplitude (250–340 ms)

We found no significant group effect, *F*(1,30) = 0.15, *p* =.70, *η_p_^2^* =.00. There was a significant main effect of face type, *F*(1,30) = 122.23, *p* < .001, *η_p_^2^* =.80, and orientation, *F*(1,30) = 66.35, *p* < .001, *η_p_^2^* =.69, with larger amplitudes for inverted than upright faces. Analyses revealed no significant interaction between face type and orientation, *F*(1,30) = 1.74, *p* =.197, *η_p_^2^* =.05, and no significant group interactions including either the factors face type, and orientation, *F*s (1,30) < 1.98. Statistics for all main and interaction effects can be found in **Table S9** (**Supplementary Materials**).

For the interaction between face type, orientation and group, the Bayesian analysis revealed a BF_excl_= 4.576, indicating that there is 4.6 times higher probability for support of the null effect than the alternative hypothesis. Statistics of Bayesian analysis for group interaction effects can be found in **Table S10** (**Supplementary Materials**).

#### LPC Amplitude (400–600 ms)

The group effect failed to reach significance, *F*(1,30) = 3.78, *p* =.061, *η_p_^2^* =.11. There was a significant main effect of face type, *F*(1,30) = 135.30, *p* < .001, *η_p_^2^* =.82, and orientation, *F*(1,30) = 7.04, *p* =.013, *η_p_^2^* =.19. Analyses revealed a significant interaction between face type and orientation, *F*(1,30) = 12.58, *p* =.001, *η_p_^2^* =.29, but no significant group interactions including the factors face type, and orientation, *Fs* (1,30) < 1.21. There was a trend for an interaction between site, expression, and group, *F*(5,150) = 2.24, *p* =.055, *η_p_^2^* =.07. Statistics for all main and interaction effects can be found in **Table S11** (**Supplementary Materials**).

**Figure 8:**
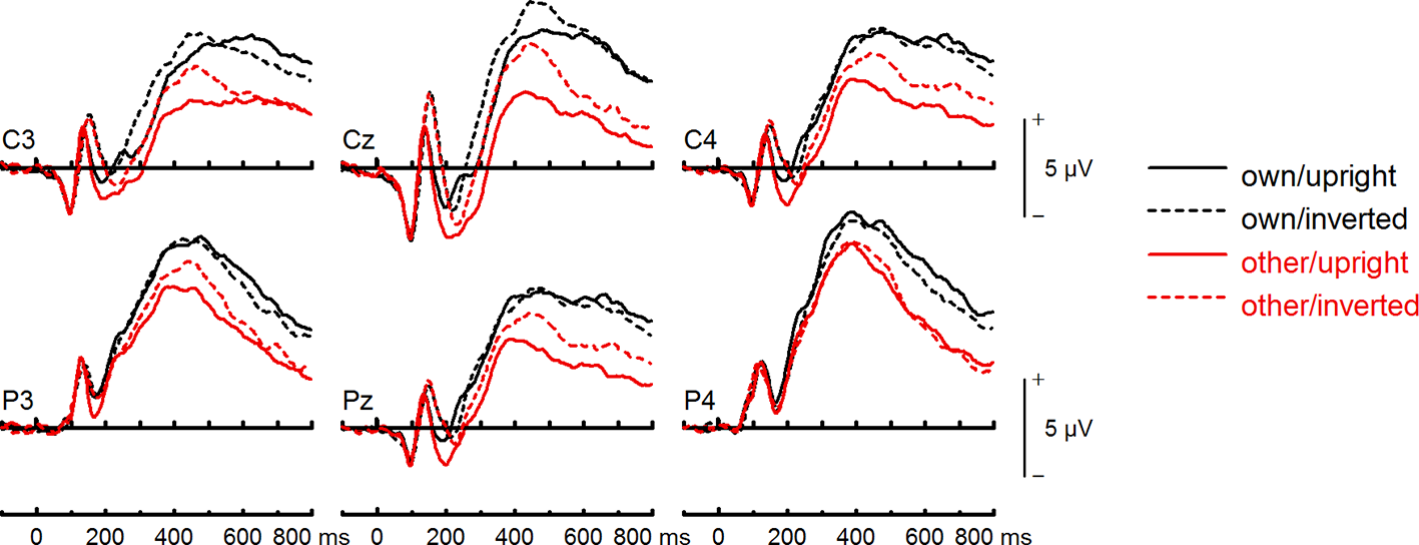
Event-related potentials (ERPs) from the test phase for the interaction face type (own vs. other) by orientation (upright vs. inverted) in body dysmorphic disorder (BDD) patients. At centro-parietal sites (C3/C4, P3/P4, Cz/Pz) within time interval for late positive component (LPC).

**Figure 9:**
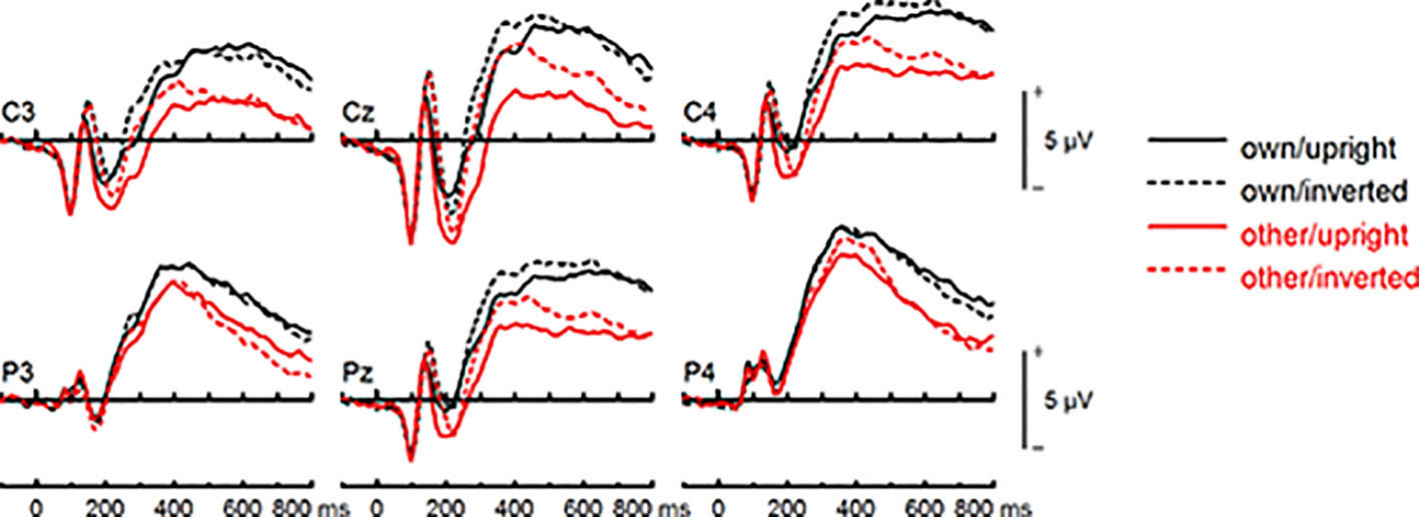
Event-related potentials (ERPs) from the test phase for the interaction face type (own vs. other) by orientation (upright vs. inverted) in healthy controls. At centro-parietal sites (C3/C4, P3/P4, Cz/Pz) within time interval for late positive component (LPC).

To further investigate the interaction trend, we performed separate ANOVAs for each group. In BDD, analyses revealed significant main effects of face type, *F*(1,15) = 105.66, *p* < .001, *η_p_^2^* =.88, and orientation, *F*(1,15) = 5.99, *p* =.027, *η_p_^2^* =.29. There was a significant interaction between face type and orientation, *F*(1,15) = 10.75, p =.005, *η*_p_^2^ =.42, indicating larger LPC inversion effects to other (relative to own) faces, with larger (i.e., more positive) LPC amplitudes for other inverted than other upright faces (see **Figures 6** and **8**). In HC, there was a significant main effect of face type, *F*(1,15) = 45.97, *p* < .001, *η_p_^2^* =.75, but no significant main effects of orientation, *F*(1,15) = 2.14, *p* =.164, *η_p_^2^* =.12, and no significant interaction between face type and orientation, *F*(1,15) = 3.01, *p* =.103, *η_p_^2^* =.17 (see **Figures 7** and **9**).

For the interaction between face type, orientation and group, the Bayesian analysis revealed a BFexcl= 2.447, indicating that there is no sufficient evidence for the absence of this null effect. Statistics of Bayesian analysis for group interaction effects can be found in **Table S12** (**Supplementary Materials**).

## Discussion

The present study is the first EEG investigation of the face inversion effect to own and other’s faces in BDD by using different ERP correlates of early visual face processing. At some variance with hypotheses and previous EEG findings in BDD (24, 25), results suggest that BDD patients in our observed sample show similarities to healthy controls both at the level of early perceptual processing of the own face (P100, N170, P200), and at the subsequent activation of neural representation of facial identity (N250) and emotional processing (LPC).

In the early P100 component, no evidence for face inversion effects was found in both groups, which is in line with previous EEG research, indicating that the P100 is not sensitive to face inversion (37). In the subsequent N170 component, both groups exhibited the common face inversion effects, with larger N170 amplitudes for inverted than upright faces, possibly reflecting a disruption of configural processing and in consequence a preponderance of featural processing in inverted faces. Contrary to our hypotheses, these effects were not reliably influenced by the factor group. In addition, no specific inversion effects to own (relative to other) faces were found in BDD, relative to controls. In the P200 component, both groups showed larger inversion effects to other (relative to own) faces, with larger P200 amplitudes for other upright than other inverted faces. Larger P200 amplitudes indicate a higher perceived typicality of a face (43, 61, 62, 71), suggesting that other upright faces were perceived as more typical and less distinctive, whereas other inverted faces were perceived as less typical and more distinctive in both groups. This overall pattern appeared to be comparable for both groups. Contrary to hypotheses, we found no smaller P200 amplitudes for own (relative to other) faces in BDD patients, relative to controls. In the N250 component, we found overall face inversion effects with larger N250 amplitudes for inverted than upright faces, which were not influenced by the factor group. Furthermore, analyses revealed no significant group differences in the processing of own (relative to other) faces, suggesting that both groups may have applied similar strategies to encode familiar and unfamiliar faces. This finding is inconsistent with previous behavioral studies that have indicated an effect of familiarity on face recognition in BDD (32, 33). In the LPC, we observed overall more positive amplitudes for other (relative to own) faces. There was also a face inversion effect with increased positivity for inverted version. This effect was more pronounced for other (relative to own) faces in both groups. The finding might reflect more efficient stimulus evaluation of upright faces due to enhanced information transmission and dominant configural processing, in particular in other faces. The *post hoc* Bayesian analysis revealed results that confirm and support the observed null effects for the analyzed ERP components. Similar to EEG findings, the results of behavioral data analyses provided no evidence for behavioral response abnormalities in BDD. For the whole group, we found a behavioral inversion effect on reaction time to own faces, as well as on accuracy to own and other’s faces. However, there was no evidence for group differences and smaller behavioral face inversion effects in BDD.

In general, the face inversion effect is mainly due to deficits in processing of configural and holistic information from inverted faces and occurs primarily at the encoding stage of face processing. Both EEG and behavioral data of the present study suggest that early configural and holistic face processing of the own face may not substantially altered in our observed BDD sample. Our findings are inconsistent with previous fMRI and behavioral studies that provided evidence for a detailed processing of facial features in own and other faces (14–17, 22) and for an abnormal processing of configural and holistic information of own and other faces, objects, scenes and bodies in BDD (23, 30–32, 72, 73). Furthermore, our data are in contrast to previous neuropsychological studies that provided preliminary evidence for smaller P100 and N170 amplitudes as markers of abnormal early structural encoding of faces, and an abnormal spatiotemporal activation of configural and holistic information in BDD (24, 25). These findings suggested an incomplete generation of a whole facial representation, which in turn may contribute to the perceptual distortions in BDD (24).

While abundant evidence exists from behavioral, fMRI and EEG studies for abnormal configural and holistic, and increased detailed processing in BDD, the findings from the current EEG study and previous behavioral studies on face inversion effect are still inconsistent. These inconsistencies might be explained by several methodological differences such as experimental conditions (e.g., presentation time of stimuli). Feusner et al. (30) failed to find a significant smaller inversion effect for short duration stimuli (500ms) but not for long duration stimuli (5,000 ms) in BDD, relative to healthy controls. Similarly, Jeffries et al. (32) provided evidence for a superior recognition of inverted famous faces for long duration stimuli (5,000 ms) in BDD, relative to healthy controls. Monzani et al. (33) examined the face inversion for short duration stimuli (between 200 and 500 ms), and found that BDD patients and healthy controls performed similarly in all aspects of holistic processing and structural encoding tested (33). Mundy and Saduski (31) found enhanced discrimination abilities for inverted faces and bodies, and higher accuracies when discriminating inverted faces and scenes for long duration stimuli (7,000 ms) in individuals with high body dysmorphic concerns, relative to healthy controls. Similar to our EEG study with a presentation time of 1,000 ms, investigations using a short presentation time between 250 and 500 ms (30, 33) failed to find smaller face inversion effects, suggesting that BDD individuals and healthy controls may equally process faces in a global, holistic and configural way when given a brief presentation (30). Thus, long presentation times between 5 and 7 s may allow more time for encoding details (30–32). This might also partly explain the results from previous fMRI and EEG studies, which found abnormal brain activation patterns during encoding of detailed and configural features for longer stimulus presentation times between 3 and 4 s (22, 23, 72), and abnormal early perceptual processing during structural encoding for presentation times of 2s (24, 25).

Furthermore, the inconsistencies in results might also be partly attributed to other methodological differences that generally limit comparisons across studies on visual processing (33) and allow only limited conclusions about abnormal early neurocognitive processes in BDD. For instance, these inconsistencies may also arise from stimulus-related issues and testing conditions. In our study, stimuli were not spatial frequency filtered, while in both EEG studies and in Feusner et al.’s studies (22, 23) facial stimuli were spatial frequency filtered. It has been suggested that different levels of spatial frequencies in stimuli convey different types of information for visual processing (24). This might have had an impact on the results. For instance, in the EEG study by Li et al. (25), BDD individuals demonstrated a hypoactivity in early visual processing regions such as the lateral occipital cortex (linked to the N170 component) for low spatial frequency (i.e., low-detail) faces, and a hyperactivity in the temporal fusiform cortex (linked to the N170 component) for high spatial frequency (i.e., high-detail) houses. However, no abnormalities were found for normal spatial frequency images, regardless of stimulus type (faces or houses). In the fMRI study by Feusner et al. (23), a hypoactivity in the lateral occipital cortex was only found for low spatial frequency own faces in BDD. Furthermore, several previous studies used simultaneous matching tasks of face or house pairs (23–25), while in the current study faces were presented in a sequential task. Long and simultaneous presentations may allow a more comparative feature-based scanning, which is less feasible during short and sequential tasks (33). All these methodological differences raise the question whether the perceptual abnormalities in BDD result from biases for detailed information due to different task conditions or stimulus-related issues, or result from a deficit in configural and holistic processing.

Therefore, further research is necessary to replicate our findings and previous EEG findings (24, 25), and to further investigate early neurocognitive processes in BDD. Overall then, it should be appreciated that the present study investigated ERP correlates of own-face perception in the context of a specific experimental paradigm, and that a comprehensive test of own-face perception across a systematically varied range of experimental conditions was beyond the scope of this single experiment. The current study has several limitations that should be considered when interpreting our findings. First, due to the relatively small sample size in both groups the beta risk was high. For this reason, we computed *post hoc* Bayesian analyses to test evidence for null results. However, a replication of the study and increasing sample size is necessary to overcome this shortcoming. Second, we did not include a clinical control group such as patients with eating disorders that share many clinical features (e.g., body image distortions) with BDD. Therefore, it is not possible to conclude whether the neurocognitive processes tested can be primarily attributed to BDD-specific encoding processes. Third, due to the inclusion of BDD patients with a moderate severity, our results are not generalizable to BDD patients with a higher severity and stronger impairments. Fourth, we did not obtain specific measures to obtain delusional beliefs and insight. Thus, we were not able to determine associations between neural signatures and poor insight in BDD. Fifth, our sample included primarily female BDD patients.

## Data Availability Statement

All datasets generated for this study are included in the article/**Supplementary Material**.

## Ethics Statement

The studies involving human participants were reviewed and approved by Ethics Committee of the Medical Faculty of the Goethe University Frankfurt (GZ 39/11). The patients/participants provided their written informed consent to participate in this study. Written informed consent was obtained from the individual(s) for the publication of any potentially identifiable images or data included in this article.

## Author Contributions

VR: recruitment and conducting clinical interviews. VR, JMK, HW, SRS, and US: study the design. HW and SRS: programming of experiment, experimental setup. FK: recruitment, stimulus preparation, and EEG acquisition. JMK, VR, HW, and SRS: data collection and data analyses. VR, JMK and SRS: data interpretation. JMK: figures. VR, JMK, HW, US and SRS: literature research and writing of publication.

## Funding

This study was supported by a grant of the German Research Foundation (DFG, Deutsche Forschungsgemeinschaft) to VR, US, and SRS (Reference No.: STA 512/9-1, RI 2571/1-2).

## Conflict of Interest

The authors declare that the research was conducted in the absence of any commercial or financial relationships that could be construed as a potential conflict of interest.
