# Supplementary material for: Neural Correlates of Own- and Other-Face Perception in Body Dysmorphic Disorder
Source: Front Psychiatry. 2020 Apr 24;11:302. doi: 10.3389/fpsyt.2020.00302 (PMC7196670; doi:10.3389/fpsyt.2020.00302)
Supplement: Supplementary file 1 [file DataSheet_1.docx]

**Supplementary Material**

**Table S1.** Statistics of Bayesian Analysis for main and interaction effects in reaction times.

| **Bayesian Analysis of Effects** | | | | | | | |
| --- | --- | --- | --- | --- | --- | --- | --- |
| **Effects** | | **P(incl)** | | **P(incl\|data)** | | **BF _excl_** | |
| Face type |  | 0.263 |  | 0.085 |  | 5.011 |  |
| Orientation |  | 0.263 |  | 0.152 |  | 2.259 |  |
| Group |  | 0.263 |  | 0.449 |  | 0.843 |  |
| Face  type ✻  Orientation |  | 0.263 |  | 0.469 |  | 0.077 |  |
| Face  type ✻  Group |  | 0.263 |  | 0.073 |  | 4.114 |  |
| Orientation  ✻  Group |  | 0.263 |  | 0.107 |  | 2.962 |  |
| Face  type ✻  Orientation  ✻  Group |  | 0.053 |  | 0.008 |  | 1.925 |  |
|  | | | | | | | |
| *Note.* Compares models that contain the effect to equivalent models stripped of the effect. Higher-order interactions are excluded. Analysis suggested by Sebastiaan Mathôt. | | | | | | | |

**Table S2.** Statistics of Bayesian Analysis for main and interaction effects in accuracies.

| **Bayesian Analysis of Effects** | | | | | | | |
| --- | --- | --- | --- | --- | --- | --- | --- |
| **Effects** | | **P(incl)** | | **P(incl\|data)** | | **BF _excl_** | |
| Face type |  | 0.263 |  | 0.033 |  | 5.257 |  |
| Orientation |  | 0.263 |  | 0.034 |  | 5.048 |  |
| Group |  | 0.263 |  | 0.264 |  | 2.245 |  |
| Face  type ✻  Orientation |  | 0.263 |  | 0.775 |  | 0.008 |  |
| Face  type ✻  Group |  | 0.263 |  | 0.075 |  | 3.568 |  |
| Orientation  ✻  Group |  | 0.263 |  | 0.070 |  | 3.850 |  |
| Face  type ✻  Orientation  ✻  Group |  | 0.053 |  | 0.015 |  | 1.013 |  |
|  | | | | | | | |
| *Note.* Compares models that contain the effect to equivalent models stripped of the effect. Higher-order interactions are excluded. Analysis suggested by Sebastiaan Mathôt. | | | | | | | |

**Table S3.** ANOVA statistics for main and interaction effects in P100 amplitude.

| P100 Amplitude | |
| --- | --- |
| Effect | Statistics |
| Main effect: group | *F*(1,30) = 1.68, *p* = 0.200 |
| Main effect: hemisphere | *F*(1,30) = 0.03, *p* = 0.859 |
| Main effect: face type | *F*(1,30) = 0.76, *p* = 0.392 |
| Main effect: orientation | *F*(1,30) = 0.07, *p* = 0.789 |
| Main effect: expression | *F*(1,30) = 0.65, *p* = 0.426 |
| Interaction effect: hemisphere x group | *F*(1,30) = 0.31, *p* = 0.582 |
| Interaction effect: face type x group | *F*(1,30) = 1.84, *p* = 0.186 |
| Interaction effect: orientation x group | *F*(1,30) = 0.56, *p* = 0.462 |
| Interaction effect: expression x group  Interaction effect: hemisphere x face type | *F*(1,30) = 1.19, *p* = 0.284  *F*(1,30) = 0.09, *p* = 0.769 |
| Interaction effect: hemisphere x face type x group | *F*(1,30) = 0.17, *p* = 0.680 |
| Interaction effect: hemisphere x orientation | *F*(1,30) = 0.05, *p* = 0.834 |
| Interaction effect: hemisphere x orientation x group | *F*(1,30) = 0.98, *p* = 0.330 |
| Interaction effect: face type x orientation | *F*(1,30) = 0.20, *p* = 0.657 |
| Interaction effect: face type x orientation x group | *F*(1,30) = 0.28, *p* = 0.602 |
| Interaction effect: hemisphere x face type x orientation | *F*(1,30) = 5.85, *p* = 0.022 |
| Interaction effect: hemisphere x face type x orientation x group | *F*(1,30) = 0.82, *p* = 0.371 |
| Interaction effect: hemisphere x expression | *F*(1,30) = 0.27, *p* = 0.607 |
| Interaction effect: hemisphere x expression x group | *F*(1,30) = 9.64, *p* = 0.004 |
| Interaction effect: face type by expression | *F*(1,30) = 3.85, *p* = 0.059 |
| Interaction effect: face type x expression x group | *F*(1,30) = 3.79, *p* = 0.061 |
| Interaction effect: hemisphere x face type x expression | *F*(1,30) = 6.65, *p* = 0.015 |
| Interaction effect: hemisphere x face type x expression x group | *F*(1,30) = 0.06, *p* = 0.800 |
| Interaction effect: orientation x expression | *F*(1,30) = 0.96, *p* = 0.336 |
| Interaction effect: orientation x expression x group | *F*(1,30) = 1.45, *p* = 0.238 |
| Interaction effect: hemisphere x orientation x expression | *F*(1,30) = 0.02, *p* = 0.901 |
| Interaction effect: hemisphere x orientation x expression x group | *F*(1,30) = 1.58, *p* = 0.219 |
| Interaction effect: face type x orientation x expression | *F*(1,30) = 1.47, *p* = 0.235 |
| Interaction effect: face type x orientation x expression x group | *F*(1,30) = 0.12, *p* = 0.735 |
| Interaction effect: hemisphere x face type x orientation x expression | *F*(1,30) = 1.10, *p* = 0.303 |
| Interaction effect: hemisphere x face type x orientation x expression x group | *F*(1,30) = 0.30, *p* = 0.587 |

**Table S4.** Statistics of Bayesian Analysis for group interaction effects in P100 amplitude.

| **Bayesian Analysis of Effects** | | | | | | | |
| --- | --- | --- | --- | --- | --- | --- | --- |
| **Effects** | | **P(incl)** | | **P(incl\|data)** | | **BF _excl_** | |
| Face  type ✻  Group |  | 0.071 |  | 0.128 |  | 6.167 |  |
| Orientation  ✻  Group |  | 0.071 |  | 0.147 |  | 5.291 |  |
| Hemisphere  ✻  Face  type ✻  Group |  | 0.298 |  | 0.032 |  | 5.439 |  |
| Hemisphere  ✻  Orientation  ✻  Group |  | 0.298 |  | 0.041 |  | 4.476 |  |
| Face  type ✻  Orientation  ✻  Group |  | 0.298 |  | 0.009 |  | 5.260 |  |
| Face  type ✻  Expression  ✻  Group |  | 0.298 |  | 0.052 |  | 3.043 |  |
| Orientation  ✻  Expression  ✻  Group |  | 0.298 |  | 0.041 |  | 4.352 |  |
| Hemisphere  ✻  Face  type ✻  Orientation  ✻  Group |  | 0.155 |  | 2.563e -4 |  | 1.142 |  |
| Hemisphere  ✻  Face  type ✻  Expression  ✻  Group |  | 0.226 |  | 0.002 |  | 5.370 |  |
| Hemisphere  ✻  Orientation  ✻  Expression  ✻  Group |  | 0.226 |  | 0.002 |  | 5.977 |  |
| Face  type ✻  Orientation  ✻  Expression  ✻  Group |  | 0.155 |  | 7.915e -5 |  | 6.416 |  |
| Hemisphere  ✻  Face  type ✻  Orientation  ✻  Expression  ✻  Group |  | 0.012 |  | 3.023e -8 |  | 1.280 |  |
|  | | | | | | | |
| *Note.* Compares models that contain the effect to equivalent models stripped of the effect. Higher-order interactions are excluded. Analysis suggested by Sebastiaan Mathôt. | | | | | | | |

**Table S5.** ANOVA statistics for main and interaction effects in N170 amplitude.

| N170 Amplitude | |
| --- | --- |
| Effect | Statistics |
| Main effect: group | *F*(1,30) = 0.14, *p* = 0.710 |
| Main effect: hemisphere | *F*(1,30) = 1.73, *p* = 0.198 |
| Main effect: site | *F*(2,60) = 2.89, *p* = 0.086 |
| Main effect: face type | *F*(1,30) = 4.25, *p* = 0.048 |
| Main effect: orientation | *F*(1,30) = 6.55, *p* = 0.016 |
| Main effect: expression | *F*(1,30) = 0.05, *p* = 0.816 |
| Interaction effect: hemisphere x group | *F*(1,30) = 3.14, *p* = 0.087 |
| Interaction effect: site x group | *F*(2,60) = 6.92, *p* = 0.007 |
| Interaction effect: face type x group | *F*(1,30) = 0.52, *p* = 0.478 |
| Interaction effect: orientation x group | *F*(1,30) = 0.07, *p* = 0.786 |
| Interaction effect: expression x group | *F*(1,30) = 0.45, *p* = 0.505 |
| Interaction effect: site x hemisphere | *F*(2,60) = 1.09, *p* = 0.330 |
| Interaction effect: site x hemisphere x group | *F(*2,60) = 1.42*, p =* 0.250 |
| Interaction effect: hemisphere x face type | *F*(1,30) = 2.66, *p* = 0.113 |
| Interaction effect: hemisphere x face type x group | *F*(1,30) = 0.40, *p* = 0.531 |
| Interaction effect: site x face type | *F(*2,60) = 0.17*, p =* 0.789 |
| Interaction effect: site x face type x group | *F(*2,60) = 2.46*, p =* 0.109 |
| Interaction effect: site x hemisphere x face type | *F*(2,60) = 0.56, *p* = 0.563 |
| Interaction effect: site x hemisphere x face type x group | *F*(2,60) = 0.77, *p* = 0.457 |
| Interaction effect: hemisphere x orientation | *F*(1,30) = 2.42, *p* = 0.130 |
| Interaction effect: hemisphere x orientation x group | *F*(1,30) = 0.08, *p* = 0.782 |
| Interaction effect: site x orientation | *F(*2,60) = 10.19*, p =* 0.000 |
| Interaction effect: site x orientation x group | *F(*2,60) = 1.51*, p =* 0.231 |
| Interaction effect: site x hemisphere x orientation | *F(*2,60) = 0.47*, p =* 0.603 |
| Interaction effect: site x hemisphere x orientation x group | F(2,60) = 0.57, p = 0.544 |
| Interaction effect: face type x orientation | *F*(1,30) = 1.46, *p* = 0.237 |
| Interaction effect: face type x orientation x group | *F*(1,30) = 2.01, *p* = 0.166 |
| Interaction effect: hemisphere x face type x orientation | *F*(1,30) = 3.99, *p* = 0.055 |
| Interaction effect: hemisphere x face type x orientation x group | *F*(1,30) = 1.20, *p =* 0.281 |
| Interaction effect: site x face type x orientation x group | *F*(2,60) = 3.31, *p* = 0.058 |
| Interaction effect: site x hemisphere x face type x orientation x group | *F*(2,60) = 0.78, *p* = 0.464 |
| Interaction effect: hemisphere x expression | *F*(1,30) = 0.06, *p* = 0.815 |
| Interaction effect: hemisphere x expression x group | *F*(1,30) = 0.08, *p* = 0.785 |
| Interaction effect: site x expression | *F*(2,60) = 0.55, *p* = 0.525 |
| Interaction effect: site x expression x group | *F*(2,60) = 0.57, *p* = 0.516 |
| Interaction effect: site x hemisphere x expression | *F*(2,60) = 1.06, *p* = 0.351 |
| Interaction effect: site x hemisphere x expression x group | *F*(2,60) = 0.42, *p* = 0.657 |
| Interaction effect: face type x expression | *F*(1,30) = 0.17, *p* = 0.686 |
| Interaction effect: face type x expression x group | *F*(1,30) = 0.11, *p* = 0.737 |
| Interaction effect: hemisphere x face type x expression | *F*(1,30) = 0.07, *p* = 0.789 |
| Interaction effect: hemisphere x face type x expression x group | *F*(1,30) = 0.36, *p* = 0.552 |
| Interaction effect: site x face type x expression | *F(*2,60) = 0.88*, p =* 0.404 |
| Interaction effect: site x face type x expression x group | *F(*2,60) = 0.82*, p =* 0.425 |
| Interaction effect: site x hemisphere x face type x expression | *F(*2,60) = 0.77*, p =* 0.467 |
| Interaction effect: site x hemisphere x face type x expression x group | *F(*2,60) = 0.72*, p =* 0.491 |
| Interaction effect: orientation x expression | *F*(1,30) = 0.11, *p* = 0.746 |
| Interaction effect: orientation x expression x group | *F*(1,30) = 0.00, *p* = 0.959 |
| Interaction effect: hemisphere x orientation x expression | *F*(1,30) = 0.05, *p* = 0.826 |
| Interaction effect: hemisphere x orientation x expression x group | *F*(1,30) = 0.13, *p* = 0.719 |
| Interaction effect: site x orientation x expression | *F(*2,60) = 2.19*, p =* 0.120 |
| Interaction effect: site x orientation x expression x group | *F(*2,60) = 0.09*, p =* 0.910 |
| Interaction effect: site x hemisphere x orientation x expression | *F(*2,60) = 0.06*, p =* 0.934 |
| Interaction effect: site x hemisphere x orientation x expression x group | *F(*2,60) = 0.87*, p =* 0.418 |
| Interaction effect: face type x orientation x expression | *F*(1,30) = 8.44, *p* = 0.007 |
| Interaction effect: face type x orientation x expression x group | *F*(1,30) = 2.64, *p* = 0.115 |
| Interaction effect: hemisphere x face type x orientation x expression | *F*(1,30) = 0.02, *p* = 0.893 |
| Interaction effect: hemisphere x face type x orientation x expression x group | *F*(1,30) = 0.11, *p* = 0.739 |
| Interaction effect: site x face type x orientation x expression | *F(*2,60) = 0.50*, p =* 0.591 |
| Interaction effect: site x face type x orientation x expression x group | *F(*2,60) = 0.27*, p =* 0.740 |
| Interaction effect: site x hemisphere x face type x orientation x expression | *F(*2,60) = 0.45*, p =* 0.643 |
| Interaction effect: site x hemisphere x face type x orientation x expression x group | *F(*2,60) = 0.59*, p =* 0.560 |

**Table S6.** Statistics of Bayesian Analysis for group interaction effects in N170 amplitude.

| **Bayesian Analysis of Effects** | | | | | | | |
| --- | --- | --- | --- | --- | --- | --- | --- |
| **Effects** | | **P(incl)** | | **P(incl\|data)** | | **BF _excl_** | |
| Face  type ✻  Group |  | 0.071 |  | 0.102 |  | 8.151 |  |
| Orientation  ✻  Group |  | 0.071 |  | 0.101 |  | 8.388 |  |
| Hemisphere  ✻  Face  type ✻  Group |  | 0.298 |  | 0.046 |  | 2.664 |  |
| Hemisphere  ✻  Orientation  ✻  Group |  | 0.298 |  | 0.022 |  | 5.897 |  |
| Face  type ✻  Orientation  ✻  Group |  | 0.298 |  | 0.006 |  | 5.282 |  |
| Face  type ✻  Expression  ✻  Group |  | 0.298 |  | 0.024 |  | 6.144 |  |
| Orientation  ✻  Expression  ✻  Group |  | 0.298 |  | 0.031 |  | 3.988 |  |
| Hemisphere  ✻  Face  type ✻  Orientation  ✻  Group |  | 0.155 |  | 4.136e -5 |  | 3.320 |  |
| Hemisphere  ✻  Face  type ✻  Expression  ✻  Group |  | 0.226 |  | 0.001 |  | 5.261 |  |
| Hemisphere  ✻  Orientation  ✻  Expression  ✻  Group |  | 0.226 |  | 9.315e -4 |  | 4.065 |  |
| Face  type ✻  Orientation  ✻  Expression  ✻  Group |  | 0.155 |  | 1.001e -4 |  | 2.261 |  |
| Hemisphere  ✻  Face  type ✻  Orientation  ✻  Expression  ✻  Group |  | 0.012 |  | 4.396e -9 |  | 6.909 |  |
|  | | | | | | | |
| *Note.* Compares models that contain the effect to equivalent models stripped of the effect. Higher-order interactions are excluded. Analysis suggested by Sebastiaan Mathôt. | | | | | | | |

**Table S7.** ANOVA statistics for main and interaction effects in P200 amplitude.

| P200 Amplitude | |
| --- | --- |
| Effect | Statistics |
| Main effect: group | *F*(1,30) = 0.37, *p* = 0.55 |
| Main effect: hemisphere | *F*(1,30) = 0.19, *p* = 0.665 |
| Main effect: site | *F*(2,60) = 74.01, *p* < 0.001 |
| Main effect: face type | *F*(1,30) = 46.09, *p* < 0.001 |
| Main effect: orientation | *F*(1,30) = 19.38, *p* < 0.001 |
| Main effect: expression | *F*(1,30) = 6.63, *p* = 0.015 |
| Interaction effect: hemisphere x group | *F*(1,30) = 1.13, *p* = 0.297 |
| Interaction effect: site x group | *F*(2,60) = 0.06, *p* = 0.865 |
| Interaction effect: face type x group | *F*(1,30) = 1.99, *p* = 0.169 |
| Interaction effect: orientation x group | *F*(1,30) = 0.09, *p* = 0.763 |
| Interaction effect: expression x group | *F*(1,30) = 1.72, *p* = 0.200 |
| Interaction effect: site x hemisphere | *F*(2,60) = 0.23, *p* = 0.730 |
| Interaction effect: site x hemisphere x group | *F*(2,60) = 0.40, *p* = 0.614 |
| Interaction effect: site x face type | *F*(2,60) = 7.99, *p* = 0.008 |
| Interaction effect: site x face type x group | *F*(2,60) = 0.49, *p* = 0.536 |
| Interaction effect: hemisphere x face type | *F*(1,30) = 2.78, *p* = 0.106 |
| Interaction effect: hemisphere x face type x group | *F*(1,30) = 0.01, *p* = 0.917 |
| Interaction effect: site x hemisphere x face type | *F*(2,60) = 6.33, *p* = 0.005 |
| Interaction effect: site x hemisphere x face type x group | *F*(2,60) = 0.01, *p* = 0.985 |
| Interaction effect: site x orientation | *F*(2,60) = 7.72, *p* = 0.006 |
| Interaction effect: site x orientation x group | *F*(2,60) = 0.82, *p* = 0.391 |
| Interaction effect: hemisphere x orientation | *F*(1,30) = 0.84, *p* = 0.367 |
| Interaction effect: hemisphere x orientation x group | *F*(1,30) = 1.88, *p* = 0.181 |
| Interaction effect: site x hemisphere x orientation | *F*(2,60) = 0.60, *p* = 0.507 |
| Interaction effect: site x hemisphere x orientation x group | *F*(2,60) = 0.17, *p* = 0.783 |
| Interaction effect: face type x orientation | *F*(1,30) = 27.24, *p* < 0.001 |
| Interaction effect: face type x orientation x group | *F*(1,30) = 0.61, *p* = 0.442 |
| Interaction effect: site x face type x orientation | *F*(2,60) = 4.95, *p* = 0.018 |
| Interaction effect: site x face type x orientation x group | *F*(2,60) = 0.28, *p* = 0.695 |
| Interaction effect: hemisphere x face type x orientation | *F*(1,30) = 0.10, *p* = 0.749 |
| Interaction effect: hemisphere x face type x orientation x group | *F*(1,30) = 0.16, *p* = 0.688 |
| Interaction effect: site x hemisphere x face type x orientation | *F*(2,60) = 2.05, *p* = 0.138 |
| Interaction effect: site x hemisphere x face type x orientation x group | *F*(2,60) = 0.63, *p* = 0.533 |
| Interaction effect: site x expression | *F*(2,60) = 1.25, *p* = 0.287 |
| Interaction effect: site x expression x group | *F*(2,60) = 0.09, *p* = 0.857 |
| Interaction effect: hemisphere x expression | *F*(1,30) = 0.07, *p* = 0.799 |
| Interaction effect: hemisphere x expression x group | *F*(1,30) = 1.41, *p* = 0.244 |
| Interaction effect: site x hemisphere x expression | *F*(2,60) = 0.58, *p* = 0.563 |
| Interaction effect: site x hemisphere x expression x group | *F*(2,60) = 0.83, *p* = 0.440 |
| Interaction effect: face type x expression | *F*(1,30) = 1.08, *p* = 0.306 |
| Interaction effect: face type x expression x group | *F*(1,30) = 0.16, *p* = 0.690 |
| Interaction effect: site x face type x expression | *F*(2,60) = 0.02, *p* = 0.953 |
| Interaction effect: site x face type x expression x group | *F*(2,60) = 0.10, *p* = 0.854 |
| Interaction effect: hemisphere x face type x expression | *F*(1,30) = 0.12, *p* = 0.728 |
| Interaction effect: hemisphere x face type x expression x group | *F*(1,30) = 0.35, *p* = 0.556 |
| Interaction effect: site x hemisphere x face type x expression | *F*(2,60) = 2.46, *p* = 0.094 |
| Interaction effect: site x hemisphere x face type x expression x group | *F*(2,60) = 0.18, *p* = 0.832 |
| Interaction effect: orientation x expression | *F*(1,30) = 2.59, *p* = 0.118 |
| Interaction effect: orientation x expression x group | *F*(1,30) = 0.22, *p* = 0.644 |
| Interaction effect: site x orientation x expression | *F*(2,60) = 2.04, *p* = 0.139 |
| Interaction effect: site x orientation x expression x group | *F*(2,60) = 0.23, *p* = 0.799 |
| Interaction effect: hemisphere x orientation x expression | *F*(1,30) = 4.87, *p* = 0.035 |
| Interaction effect: hemisphere x orientation x expression x group | *F*(1,30) = 2.23, *p* = 0.146 |
| Interaction effect: site x hemisphere x orientation x expression | *F*(2,60) = 0.02, *p* = 0.978 |
| Interaction effect: site x hemisphere x orientation x expression x group | *F*(2,60) = 1.14, *p* = 0.326 |
| Interaction effect: face type x orientation x expression | *F*(1,30) = 0.15, *p* = 0.703 |
| Interaction effect: face type x orientation x expression x group | *F*(1,30) = 0.06, *p* = 0.813 |
| Interaction effect: site x face type x orientation x expression | *F*(2,60) = 4.96, *p* = 0.017 |
| Interaction effect: site x face type x orientation x expression x group | *F*(2,60) = 4.66, *p* = 0.021 |
| Interaction effect: hemisphere x face type x orientation x expression | *F*(1,30) = 1.10, *p* = 0.303 |
| Interaction effect: hemisphere x face type x orientation x expression x group | *F*(1,30) = 0.85, *p* = 0.364 |
| Interaction effect: site x hemisphere x face type x orientation x expression | *F*(2,60) = 2.07, *p* = 0.135 |
| Interaction effect: site x hemisphere x face type x orientation x expression x group | *F*(2,60) = 0.05, *p* = 0.955 |

**Table S8.** Statistics of Bayesian Analysis for group interaction effects in P200 amplitude.

| **Bayesian Analysis of Effects** | | | | | | | |
| --- | --- | --- | --- | --- | --- | --- | --- |
| **Effects** | | **P(incl)** | | **P(incl\|data)** | | **BF _excl_** | |
| Face  type ✻  Group |  | 0.071 |  | 0.261 |  | 2.427 |  |
| Orientation  ✻  Group |  | 0.071 |  | 0.099 |  | 8.247 |  |
| Hemisphere  ✻  Face  type ✻  Group |  | 0.298 |  | 0.046 |  | 6.976 |  |
| Hemisphere  ✻  Orientation  ✻  Group |  | 0.298 |  | 0.052 |  | 2.556 |  |
| Face  type ✻  Orientation  ✻  Group |  | 0.298 |  | 0.015 |  | 4.484 |  |
| Face  type ✻  Expression  ✻  Group |  | 0.298 |  | 0.055 |  | 5.557 |  |
| Orientation  ✻  Expression  ✻  Group |  | 0.298 |  | 0.030 |  | 5.107 |  |
| Hemisphere  ✻  Face  type ✻  Orientation  ✻  Group |  | 0.155 |  | 1.075e -4 |  | 4.223 |  |
| Hemisphere  ✻  Face  type✻  Expression  ✻  Group |  | 0.226 |  | 0.002 |  | 3.314 |  |
| Hemisphere  ✻  Orientation  ✻  Expression  ✻  Group |  | 0.226 |  | 0.002 |  | 3.641 |  |
| Face  type ✻  Orientation  ✻  Expression  ✻  Group |  | 0.155 |  | 6.644e -5 |  | 5.171 |  |
| Hemisphere  ✻  Face  type ✻  Orientation  ✻  Expression  ✻  Group |  | 0.012 |  | 1.189e -8 |  | 10.233 |  |
|  | | | | | | | |
| *Note.* Compares models that contain the effect to equivalent models stripped of the effect. Higher-order interactions are excluded. Analysis suggested by Sebastiaan Mathôt. | | | | | | | |

**Table S9.** ANOVA statistics for main and interaction effects in N250 amplitude.

| N250 Amplitude | |
| --- | --- |
| Effect | Statistics |
| Main effect: group | *F*(1,30) = 0.15, *p* = 0.700 |
| Main effect: hemisphere | *F*(1,30) = 11.47, *p* = 0.002 |
| Main effect: site | *F*(2,60) = 107.29, *p* < 0.001 |
| Main effect: face type | *F*(1,30) = 122.23, *p* < 0.001 |
| Main effect: orientation | *F*(1,30) = 66.35, *p* < 0.001 |
| Main effect: expression | *F*(1,30) = 4.59, *p* = 0.040 |
| Interaction effect: hemisphere x group | *F*(1,30) = 1.06, *p* = 0.311 |
| Interaction effect: site x group | *F*(2,60) = 2.19, *p* = 0.130 |
| Interaction effect: face type x group | *F*(1,30) = 0.15, *p* = 0.702 |
| Interaction effect: orientation x group | *F*(1,30) = 0.22, *p* = 0.642 |
| Interaction effect: expression x group | *F*(1,30) = 0.59, *p* = 0.450 |
| Interaction effect: site x hemisphere | *F*(2,60) = 11.75, *p* < 0.001 |
| Interaction effect: site x hemisphere x group | *F*(2,60) = 1.24, *p* = 0.289 |
| Interaction effect: hemisphere x face type | *F*(1,30) = 9.68, *p* = 0.004 |
| Interaction effect: hemisphere x face type x group | *F*(1,30) = 0.04, *p* = 0.833 |
| Interaction effect: site x face type | *F*(2,60) = 9.77, *p* = 0.001 |
| Interaction effect: site x face type x group | *F*(2,60) = 1.10, *p* = 0.323 |
| Interaction effect: site x hemisphere x face type | *F*(2,60) = 4.96, *p* = 0.01 |
| Interaction effect: site x hemisphere x face type x group | *F*(2,60) = 2.18, *p* = 0.121 |
| Interaction effect: hemisphere x orientation | *F*(1,30) = 11.28, *p* = 0.002 |
| Interaction effect: hemisphere x orientation x group | *F*(1,30) = 0.40, *p* = 0.534 |
| Interaction effect: site x orientation | *F*(2,60) = 5.96, *p* = 0.012 |
| Interaction effect: site x orientation x group | *F*(2,60) = 0.43, *p* = 0.576 |
| Interaction effect: site x hemisphere x orientation | *F*(2,60) = 1.94, *p* = 0.159 |
| Interaction effect: site x hemisphere x orientation x group | *F*(2,60) = 0.61, *p* = 0.525 |
| Interaction effect: face type x orientation | *F*(1,30) = 1.74, *p* = 0.197 |
| Interaction effect: face type x orientation x group | *F*(1,30) = 1.01, *p* = 0.323 |
| Interaction effect: hemisphere x face type x orientation | *F*(1,30) = 1.11, *p* = 0.30 |
| Interaction effect: hemisphere x face type x orientation x group | *F*(1,30) = 0.03, *p* = 0.857 |
| Interaction effect: site x face type x orientation | *F*(2,60) = 1.62, *p* = 0.211 |
| Interaction effect: site x face type x orientation x group | *F*(2,60) = 0.01, *p* = 0.979 |
| Interaction effect: site x hemisphere x face type x orientation | *F*(2,60) = 2.51, *p* = 0.090 |
| Interaction effect: site x hemisphere x face type x orientation x group | *F*(2,60) = 0.46, *p* = 0.636 |
| Interaction effect: hemisphere x expression | *F*(1,30) = 2.51, *p* = 0.124 |
| Interaction effect: hemisphere x expression x group | *F*(1,30) = 0.07, *p* = 0.794 |
| Interaction effect: site x expression | *F*(2,60) = 1.45, *p* = 0.244 |
| Interaction effect: site x expression x group | *F*(2,60) = 0.11, *p* = 0.860 |
| Interaction effect: site x hemisphere x expression | *F*(2,60) = 1.24, *p* = 0.296 |
| Interaction effect: site x hemisphere x expression x group | *F*(2,60) = 0.22, *p* = 0.802 |
| Interaction effect: face type x expression | *F*(1,30) = 0.33, *p* = 0.568 |
| Interaction effect: face type x expression x group | *F*(1,30) = 2.81, *p* = 0.104 |
| Interaction effect: hemisphere x face type x expression | *F*(1,30) = 0.19, *p* = 0.670 |
| Interaction effect: hemisphere x face type x expression x group | *F*(1,30) = 1.04, *p* = 0.316 |
| Interaction effect: site x face type x expression | *F*(2,60) = 1.19, *p* = 0.312 |
| Interaction effect: site x face type x expression x group | *F*(2,60) = 0.22, *p* = 0.804 |
| Interaction effect: site x hemisphere x face type x expression | *F*(2,60) = 1.67, *p* = 0.197 |
| Interaction effect: site x hemisphere x face type x expression x group | *F*(2,60) = 1.05, *p* = 0.356 |
| Interaction effect: orientation x expression | *F*(1,30) = 0.32, *p* = 0.578 |
| Interaction effect: orientation x expression x group | *F*(1,30) = 2.66, *p* = 0.113 |
| Interaction effect: hemisphere x orientation x expression | *F*(1,30) = 7.61, *p* = 0.010 |
| Interaction effect: hemisphere x orientation x expression x group | *F*(1,30) = 0.11, *p* = 0.745 |
| Interaction effect: site x orientation x expression | *F*(2,60) = 3.42, *p* = 0.040 |
| Interaction effect: site x orientation x expression x group | *F*(2,60) = 0.09, *p* = 0.909 |
| Interaction effect: site x hemisphere x orientation x expression | *F*(2,60) = 0.09, *p* = 0.917 |
| Interaction effect: site x hemisphere x orientation x expression x group | *F*(2,60) = 0.68, *p* = 0.510 |
| Interaction effect: face type x orientation x expression | *F*(1,30) = 0.15, *p* = 0.705 |
| Interaction effect: face type x orientation x expression x group | *F*(1,30) = 2.95, *p* = 0.096 |
| Interaction effect: hemisphere x face type x orientation x expression | *F*(1,30) = 0.87, *p* = 0.358 |
| Interaction effect: hemisphere x face type x orientation x expression x group | *F*(1,30) = 2.85, *p* = 0.102 |
| Interaction effect: site x face type x orientation x expression | *F*(2,60) = 2.00, *p* = 0.147 |
| Interaction effect: site x face type x orientation x expression x group | *F*(2,60) = 0.65, *p* = 0.520 |
| Interaction effect: site x hemisphere x face type x orientation x expression | *F*(2,60) = 0.13, *p* = 0.877 |
| Interaction effect: site x hemisphere x face type x orientation x expression x group | *F*(2,60) = 2.72, *p* = 0.074 |

**Table S10.** Statistics of Bayesian Analysis for group interaction effects in N250 amplitude.

| **Bayesian Analysis of Effects** | | | | | | | |
| --- | --- | --- | --- | --- | --- | --- | --- |
| **Effects** | | **P(incl)** | | **P(incl\|data)** | | **BF _excl_** | |
| Face  type ✻  Group |  | 0.071 |  | 0.117 |  | 6.981 |  |
| Orientation  ✻  Group |  | 0.071 |  | 0.141 |  | 5.432 |  |
| Hemisphere  ✻  Face  type ✻  Group |  | 0.298 |  | 0.028 |  | 5.542 |  |
| Hemisphere  ✻  Orientation  ✻  Group |  | 0.298 |  | 0.042 |  | 4.465 |  |
| Face  type ✻  Orientation  ✻  Group |  | 0.298 |  | 0.009 |  | 4.576 |  |
| Face  type ✻  Expression  ✻  Group |  | 0.298 |  | 0.036 |  | 4.028 |  |
| Orientation  ✻  Expression  ✻  Group |  | 0.298 |  | 0.048 |  | 3.769 |  |
| Hemisphere  ✻  Face  type ✻  Orientation  ✻  Group |  | 0.155 |  | 5.886e -5 |  | 4.170 |  |
| Hemisphere  ✻  Face  type ✻  Expression  ✻  Group |  | 0.226 |  | 0.001 |  | 3.209 |  |
| Hemisphere  ✻  Orientation  ✻  Expression  ✻  Group |  | 0.226 |  | 0.001 |  | 5.072 |  |
| Face  type ✻  Orientation  ✻  Expression  ✻  Group |  | 0.155 |  | 8.560e -5 |  | 5.374 |  |
| Hemisphere  ✻  Face  type ✻  Orientation  ✻  Expression  ✻  Group |  | 0.012 |  | 1.028e -8 |  | 3.443 |  |
|  | | | | | | | |
| *Note.*  Compares models that contain the effect to equivalent models stripped of the effect. Higher-order interactions are excluded. Analysis suggested by Sebastiaan Mathôt. | | | | | | | |

**Table S11.** ANOVA statistics for main and interaction effects in LPC amplitude.

| LPC Amplitude | |
| --- | --- |
| Effect | Statistics |
| Main effect: group | *F*(1,30) = 3.78, *p* = 0.061 |
| Main effect: site | *F*(5,150) = 26.58, *p* < 0.001 |
| Main effect: face type | *F*(1,30) = 135.30, *p* < 0.001 |
| Main effect: orientation | *F*(1,30) = 7.04, *p* = 0.013 |
| Main effect: expression | *F*(1,30) = 0.89, *p* = 0.353 |
| Interaction effect: site x group | *F*(5,150) = 1.25, *p* = 0.289 |
| Interaction effect: face type x group | *F*(1,30) = 0.75, *p* = 0.394 |
| Interaction effect: orientation x group | *F*(1,30) = 0.11, *p* = 0.738 |
| Interaction effect: expression x group | *F*(1,30) = 2.65, *p* = 0.114 |
| Interaction effect: site x face type | *F*(5,150) = 6.03, *p* < 0.001 |
| Interaction effect: site x face type x group | *F*(5,150) = 0.91, *p* = 0.479 |
| Interaction effect: site x orientation | *F*(5,150) = 4.23, *p* = 0.001 |
| Interaction effect: site x orientation x group | *F*(5,150) = 0.63, *p* = 0.677 |
| Interaction effect: face type x orientation | *F*(1,30) = 12.58, *p* = 0.001 |
| Interaction effect: face type x orientation x group | *F*(1,30) = 1.21, *p* = 0.281 |
| Interaction effect: site x face type x orientation | *F*(5,150) = 2.58, *p* = 0.028 |
| Interaction effect: site x face type x orientation x group | *F*(5,150) = 1.66, *p* = 0.148 |
| Interaction effect: site x expression | *F*(5,150) = 0.66, *p* = 0.651 |
| Interaction effect: site x expression x group | *F*(5,150) = 2.24, *p* = 0.054 |
| Interaction effect: face type x expression | *F*(1,30) = 0.44, *p* = 0.515 |
| Interaction effect: face type x expression x group | *F*(1,30) = 1.75, *p* = 0.195 |
| Interaction effect: site x face type x expression | *F*(5,150) = 1.22, *p* = 0.303 |
| Interaction effect: site x face type x expression x group | *F*(5,150) = 1.06, *p* = 0.386 |
| Interaction effect: orientation x expression | *F*(1,30) = 3.05, *p* = 0.091 |
| Interaction effect: orientation x expression x group | *F*(1,30) = 0.15, *p* = 0.697 |
| Interaction effect : site x orientation x expression | *F*(5,150) = 1.34, *p* = 0.250 |
| Interaction effect : site x orientation x expression x group | *F*(5,150) = 0.67, *p* = 0.646 |
| Interaction effect: face type x orientation x expression | *F*(1,30) = 0.41, *p* = 0.527 |
| Interaction effect: face type x orientation x expression x group | *F*(1,30) = 0.60, *p* = 0.445 |
| Interaction effect: site x face type x orientation x expression | *F*(5,150) = 1.08, *p* = 0.375 |
| Interaction effect: site x face type x orientation x expression x group | *F*(5,150) = 0.39, *p* = 0.856 |

**Table S12.** Statistics of Bayesian Analysis for group interaction effects in LPC amplitude.

| **Bayesian Analysis of Effects** | | | | | | | |
| --- | --- | --- | --- | --- | --- | --- | --- |
| **Effects** | | **P(incl)** | | **P(incl\|data)** | | **BF _excl_** | |
| Face  type ✻  Group |  | 0.071 |  | 0.202 |  | 3.377 |  |
| Orientation  ✻  Group |  | 0.071 |  | 0.125 |  | 6.535 |  |
| Hemisphere  ✻  Face  type ✻  Group |  | 0.298 |  | 0.014 |  | 21.405 |  |
| Hemisphere  ✻  Orientation  ✻  Group |  | 0.298 |  | 0.023 |  | 6.787 |  |
| Face  type ✻  Orientation  ✻  Group |  | 0.298 |  | 0.016 |  | 2.447 |  |
| Face  type ✻  Expression  ✻  Group |  | 0.298 |  | 0.091 |  | 2.483 |  |
| Orientation  ✻  Expression  ✻  Group |  | 0.298 |  | 0.022 |  | 7.303 |  |
| Hemisphere  ✻  Face  type ✻  Orientation  ✻  Group |  | 0.155 |  | 9.398e  -6 |  | 6.720 |  |
| Hemisphere  ✻  Face  type ✻  Expression  ✻  Group |  | 0.226 |  | 2.985e  -4 |  | 9.968 |  |
| Hemisphere  ✻  Orientation  ✻  Expression  ✻  Group |  | 0.226 |  | 2.157e  -4 |  | 10.534 |  |
| Face  type ✻  Orientation  ✻  Expression  ✻  Group |  | 0.155 |  | 8.890e  -5 |  | 1.690 |  |
| Hemisphere  ✻  Face  type ✻  Orientation  ✻  Expression  ✻  Group |  | 0.012 |  | 7.979e -11 |  | 6.525 |  |
|  | | | | | | | |
| *Note.*  Compares models that contain the effect to equivalent models stripped of the effect. Higher-order interactions are excluded. Analysis suggested by Sebastiaan Mathôt. | | | | | | | |
